# Non-apoptotic activity of the mitochondrial protein SMAC/Diablo in lung cancer: Novel target to disrupt survival, inflammation, and immunosuppression

**DOI:** 10.3389/fonc.2022.992260

**Published:** 2022-09-14

**Authors:** Swaroop Kumar Pandey, Anna Shteinfer-Kuzmine, Vered Chalifa-Caspi, Varda Shoshan-Barmatz

**Affiliations:** ^1^ The National Institute for Biotechnology in the Negev, Ben-Gurion University of the Negev, Beer-Sheva, Israel; ^2^ Ilse Katz Institute for Nanoscale Science & Technology, Ben-Gurion University of the Negev, Beer-Sheva, Israel; ^3^ Department of Life Sciences, Ben-Gurion University of the Negev, Beer-Sheva, Israel

**Keywords:** SMAC, cell proliferation, inflammation, lung cancer, immunosuppression

## Abstract

Mitochondrial SMAC/Diablo induces apoptosis by binding the inhibitor of apoptosis proteins (IAPs), thereby activating caspases and, subsequently, apoptosis. Previously, we found that despite its pro-apoptotic activity, SMAC/Diablo is overexpressed in cancer, and demonstrated that in cancer it possesses new essential and non-apoptotic functions that are associated with regulating phospholipid synthesis including modulating mitochondrial phosphatidylserine decarboxylase activity. Here, we demonstrate additional functions for SMAC/Diablo associated with inflammation and immunity. CRISPR/Cas9 SMAC/Diablo-depleted A549 lung cancer cells displayed inhibited cell proliferation and migration. Proteomics analysis of these cells revealed altered expression of proteins associated with lipids synthesis and signaling, vesicular transport and trafficking, metabolism, epigenetics, the extracellular matrix, cell signaling, and neutrophil-mediated immunity. SMAC-KO A549 cell-showed inhibited tumor growth and proliferation and activated apoptosis. The small SMAC-depleted “tumor” showed a morphology of alveoli-like structures, reversed epithelial-mesenchymal transition, and altered tumor microenvironment. The SMAC-lacking tumor showed reduced expression of inflammation-related proteins such as NF-kB and TNF-α, and of the PD-L1, associated with immune system suppression. These results suggest that SMAC is involved in multiple processes that are essential for tumor growth and progression. Thus, targeting SMAC’s non-canonical function is a potential strategy to treat cancer.

## Introduction

SMAC/Diablo, the second mitochondrial-derived activator of caspases (SMAC) is also known as a direct inhibitor of apoptosis-binding low pI (DIABLO). SMAC is produced and a precursor and resides within the intermembranous space (IMS) of the mitochondria (1). Upon apoptosis initiation, proteolytical cleavage of the SMAC/DIABLO (SMAC) mitochondrial targeting signal takes place before its translocation from the mitochondria into the cytosol. SMAC, as other proapoptotic proteins (Cyto *c*, AIF, Htra2, Endo-G), is released to the cytosol *via* a protein-conducting channel assembled in the outer mitochondrial membrane in response to an apoptotic signal (2). The released SMAC interacts with inhibitors of apoptosis proteins (IAPs) and competes with caspase-3 and -9 for binding to IAPs, allowing the activation of the caspase cascade, and thereby, induction of apoptosis (3–5). XIAP, an important member of IAPs, was shown to interact with SMAC with a stoichiometric inhibitory interaction of one XIAP homodimer binds to one tetramer of SMAC, forming a 201.5 kDa complex (6).

Thus, SMAC, by inhibiting IAPs’ anti-apoptotic function promotes apoptotic cell death.

One of the hallmarks of cancer is deregulated apoptosis (7), involving several mechanisms including overexpression of IAP and failure of the IAP antagonist SMAC to translocate from the mitochondria to the cytosol (8). IAPs expression levels were increased in a number of human tumor and their overexpression has been correlated with tumor growth, and poor prognosis or low response to treatment (9).

However, IAPs not only regulate caspases and apoptosis, but also modulate inflammatory signaling and immunity, mitogenic kinase signaling, proliferation and mitosis, as well as cell invasion and metastasis (10). Expressing full-length SMAC (11), pro-SMAC (8), or cytosol-targeted SMAC (tSMAC), (12) showed that SMAC plays a pivotal role in the onset of cancer cell apoptosis. However, another study demonstrated that SMAC does not induce apoptosis by itself, even when expressed as a mature form in the cytosol of mammalian cells (4). Moreover, disruption of the *Smac* gene in mice produces no obvious phenotype—the mice are viable, and grow and mature normally, without any histological abnormalities, and exhibit wild-type responses to all types of apoptotic stimuli (13). However, it has been suggested that the role of SMAC can vary depending on the cell type (14).

Despite SMAC pro-apoptotic function, it is expressed in a wide range of normal tissues and in some tumor cells lines. It is overexpressed in different types of cancer (15–17), such as breast, lung, bladder, cervical, pancreas, prostate, and colorectal cancer, as well as melanoma and glioma (18). This SMAC overexpression in primary human tumors suggests that it possesses additional non-apoptotic functions.

Recently, we explored this non-apoptotic function of SMAC by silencing its expression using specific siRNA in cells in culture or in sub-cutaneous lung cancer xenografts in mice (19, 20). We demonstrated that SMAC regulates mitochondrial phosphatidylserine decarboxylase (PSD) activity that catalyzes the synthesis of phosphatidylethanolamine (PE) from phosphatidylserine (PS), with SMAC depletion leading to increased PE in the mitochondria, while decreasing the level of all phospholipids in the cell (20). Moreover, inhibition of PSD by specific peptides targeted to the mitochondria or to the nucleus resulted in inhibiting cell growth. These results suggest that SMAC and its associated protein PSD (20) are necessary to promote neoplastic metaplasia.

In this study, we further evaluated the function of SMAC in cancer using CRISPR/Cas9-mediated SMAC knockout (SMAC-KO) lung cancer A549 cells. Our results show that tumors comprising SMAC-lacking cells show reversed epithelial-mesenchymal transition (EMT), altered microenvironment and highly reduced expression of inflammation-related proteins and of the programmed death-ligand 1 (PD-L1), associated with suppression of the adaptive immune system. In addition, a proteomics analysis showed a larger number of the differentially expressed proteins between SMAC expressing and non-expressing cells. These include proteins associated with lipids and lipid-signaling molecules, metabolism, DNA- and RNA-associated processes, transport and intracellular trafficking, cellular signaling, immunity, and more, pointing to SMAC’s multiple functions in cancer.

## Materials and methods

Materials, the TUNEL assay, the Sulforhodamine B (SRB) cell proliferation assay, the migration and wound-healing assay, and protein extraction and immunoblots are presented in the Supplementary Materials.

### Cell culture and CRISPR/Cas9 SMAC knockout

A549 (human lung adenocarcinoma epithelial cell) cell line was purchased from the American Type Culture Collection (ATCC) (Manassas, VA). Cells were maintained in DMEM medium provided with 10% FBS at 37°C in an incubator with 5% CO_2_. Cell line was routinely tested for mycoplasma contamination.

SMAC CRISPR/Cas9 Knockout Plasmid with GFP marker (sc-402009) was purchased from Santa Cruz Biotechnology (Dallas, TX). A549 cells were seeded in 6-well cell culture plates (200,000 cells/well) and allowed to attach overnight. Cells were transfected with SMAC CRISPR/Cas9 Knockout Plasmid as per manufacturers’ instructions using JetPRIME transfection reagent. GFP-positive cells were sorted using FACS (SY3200 cell sorter; Synergy) and plated in 96-well plates (1 cell/well). Cells grew for 10 days, and each colony was transferred to a separate well of 12-well cell culture plates. Individual colonies having SMAC-KO were selected for maintenance after immunoblotting for SMAC.

### Xenograft mouse models

Female athymic nude mice (7–8 weeks old) (weight ~20–25g) were procured from Envigo and allowed a week of acclimatization to their new surroundings. Lung cancer A549 cells or SMAC CRISPR/Cas9 Knockout A549 cells (3x10**^6^**) were implanted subcutaneously on the dorsal flanks of the mice. The size of the developed tumors was monitored twice a week for a period of 33 days in two dimensions using a digital caliper, and volumes were calculated using the formula (*π*/6)*(*L* × *W* ^2^) (*L* = length; *W* = width). At the end point of the experiment, i.e., when the mice were sacrificed using CO_2_ gas, the tumors were excised and *ex-vivo* weight was determined. Half of each tumor was either fixed and processed for IHC or frozen in liquid nitrogen for later immunoblotting and RNA isolation. Approval for the experimental protocol was obtained from the Institutional Animal Care and Use Committee of the Soroka University Medical Center.

### Immunohistochemistry and Immunofluorescence

IF staining of cells was performed in cells plated on sterile glass coverslips placed in 12-well cell culture plates (30,000 cells/well) and incubated overnight in a CO_2_ incubator, washed with PBS, and fixed with 4% paraformaldehyde. To reduce non-specific binding, cells were incubated with 5% normal goat serum for 2 h, then incubated with primary antibodies (**Table S1**) overnight at 4° C. Following overnight incubation with the primary antibodies, PBST (PBS containing 0.1% Triton-X100)-washed samples were incubated with fluorescent-tagged secondary antibodies for 2 h at room temperature in the dark. Following a wash with PBS, samples were incubated with DAPI for 15 min in the dark, washed, mounted with mounting medium (Immuno bio science, Mukilteo, Washington, USA Fluoroshield ). and viewed by confocal microscopy (Olympus 1X81).

IHC and IF of tumor sections was performed on formalin-fixed and paraffin-embedded tumor sections that were deparaffinized using xylene and a series of ethanol treatments. Sections were then incubated with 3% H_2_O_2_ for 10 min to block endogenous peroxidase activity. Antigen retrieval was done in 0.01 M citrate buffer (pH 6.0) at 95–98° C for 30 min, followed by PBST wash. In order to reduce non-specific binding, sections were incubated in 10% normal goat serum for 2 h, and then incubated with primary antibodies (**Table S1**) overnight at 4°C. For IHC, after washing with PBST, sections were incubated for 2 h at room temperature with HRP-conjugated secondary antibodies, washed well with PBST, and incubated with the substrate DAB. Sections were washed with water, counterstained with hematoxylin, and mounted with mounting medium (ORSAtec GmbH, Bobingem). Sections were observed under a microscope (Leica DM2500), and images were collected at 20**×** magnification with the same light intensity and exposure time. For IF, following overnight incubation with the primary antibodies, PBST-washed sections were incubated with fluorescent-tagged secondary antibodies for 2 h at room temperature in the dark. Following a wash with PBST, sections were incubated with DAPI for 15 min in the dark, washed, mounted with mounting medium Fluoroshield (Immuno bio science, Mukilteo, Washington, USA), and viewed by confocal microscopy (Olympus 1X81).

### RNA preparation, qRT-PCRanalysis

Total RNA was isolated from cells and tumor tissues using an RNeasy mini kit (Qiagen), according to the manufacturer’s instructions. Complementary DNA (cDNA) was synthesized with a PCRBio cDNA synthesis kit (PCR Biosystems, Wayne, PA, USA) and used for real-time q-RT-PCR using specific primers (**Table S2**) with Power SYBER green master mix (Applied Biosystems, Foster City, CA). Levels of target genes were normalized relative to β-actin mRNA levels. Samples were amplified by a 7300 Real Time PCR System (Applied Biosystems) for 40 cycles using the following PCR parameters: 95° C for 15 s, 60° C for 1 min, and 72°C for 1 min. Relative expression levels for each gene in each sample were calculated by the ddCT-based calibrated standard curve method. The mean fold changes (± SEM) of the three replicates were calculated.

### Liquid chromatography–high-resolution mass spectrometry and proteomics analysis

For the LC-HR MS/MS analysis, proteins were extracted from CRISPR/Cas9 SMAC knockout cells using lysis buffer [100 mM Tris-HCl, pH 8.0, 5 mM DTT 4% SDS, and a protease inhibitor cocktail (Calbiochem)], followed by homogenization, incubation for 3 min at 95° C, and centrifugation (10 min, 15,000 g). The protein concentration of each lysate was determined using a Lowry assay. Samples were stored at -80° C until LC-HR-MS/MS analysis. Samples were subjected to tryptic digestion, alkylation, detergent removal, and desalting, and then to LC-HR MS/MS analysis, described in the Supplementary Materials section. Mass spectrometry (MS)-based proteomics profiling and initial processing of the results were carried out at the de Botton Institute for Protein Profiling, G-INCPM at the Weizmann Institute of Science.

### Statistical analyses for identification of differentially expressed proteins

MS/MS raw data were processed with MaxQuant v1.6.6.0. The data were searched with the Andromeda search engine against the human SwissProt proteome database and appended with common lab protein contaminants. Quantification and normalization were performed using the LFQ method, yielding a total of 4,968 identified proteins. This data analysis was carried out by de the Botton Institute. A subsequent bioinformatic analysis was carried out at the Bioinformatics Core Facility at Ben-Gurion University, using the R and Partek Genomics Suite.

Proteins marked as “contaminant” were filtered out. In an additional filtering step, only proteins in which at least one of the groups (SMAC KO, Control) had two non-zero replicates were retained (n=4636). LFQ intensities were Log_2_ transformed, and zero intensities were imputed (replaced) by random numbers derived from a normal distribution in the low expression range (width = 0.2, downshift = 1.8). Imputation was repeated ten times to avoid relying too heavily on fabricated numbers. Each of the ten imputed datasets was submitted for hypothesis testing for differential protein expressions using Limma (21). The statistical model tested the contrast: SMAC-KO *vs*. Control. A protein was considered differentially expressed (DE) if it had a nominal p-value < 0.05 and an absolute fold change (in linear scale) > 1.6 in at least eight of the ten imputed datasets. The analyzed proteins were selected based on human proteins for which at least one unique peptide was identified.

Hierarchical clustering of the DE proteins was performed in Partek, using Pearson’s dissimilarity and complete linkage. For hierarchical clustering, Log_2_-transformed LFQ values were z-scored after the zero values were replaced by the global minimum (19.443). Enrichment analysis versus the gene ontology (GO) biological process and GO cellular component was performed in Enrich R (22) using a Fisher’s exact test.

### Statistics

Results are presented as the means ± SEM of results obtained from independent experiments. A difference was considered statistically significant when the p-value was deemed <0.05 (*), < 0.01 (**), <0.001 (***), or ****p ≤ 0.0001 assessed through an unpaired Student’s two-tailed t-test.

## Results

### SMAC-KO A549 cells show inhibited proliferation and migration

**Figure 1:**
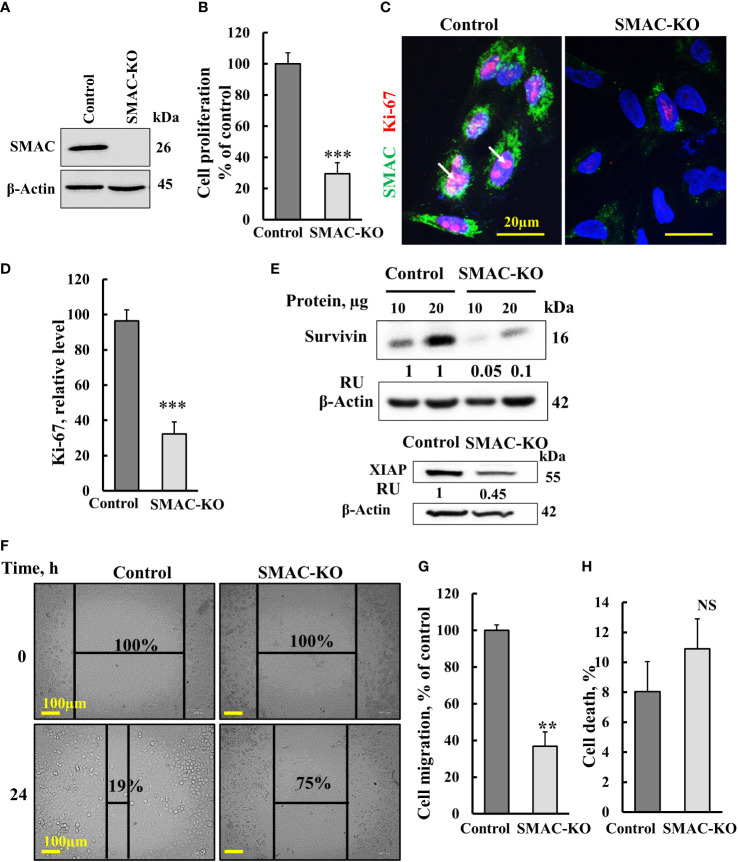
Crispr/Cas-9 SMAC knockout in A549 cells inhibits cell proliferation and migration. **(A)** Immunoblot of CRISPR/Cas9-generated SMAC-deficient A549 cells. **(B)** Cell proliferation in SMAC-KO A549 cells as analyzed using the SRB method. **(C, D)** A549 cells expressing SMAC and SMAC-KO A549 cells were IF co-stained with anti-SMAC and anti-Ki-67 antibodies **(C)** with quantitative analysis of Ki-67 expression levels **(D)**. **(E)** Immunoblot analysis of survivin and XIAP in protein extracts from SMAC-expressing and SMAK-KO cells. **(F, G),** A549 cells expressing SMAC and SMAC-KO A549 cells were allowed to grow to 80% confluence. The cell layer was scraped using a 200-µl sterile pipette tip to create a scratch/wound devoid of cells. Migration was assessed after 24 h. Representative photomicrographs are shown **(F)** (n=3). Quantification of the results describes the change in percentage of the scratch size at the indicated times (n=3) **(G)**. **(H)** Control and SMAC-KO A549 cells were analyzed for cell death using propidium iodine staining and FACS analysis. Results are the means± SEM, **p ≤ 0.01; ***p < 0.001, NS, non-significant.

Here, we studied the function of SMAC on cancer cell growth and tumor oncogenic properties using CRISPR/Cas9-mediated SMAC knockout (SMAC-KO) lung cancer A549 cells tested in culture and as a xenograft in nude mice. As expected, SMAC-KO cells showed no SMAC expression and reduced cell proliferation, as assayed by the SRB method (**Figures 1A, B**). The reduced proliferation of SMAC-KO cells was also reflected in the decreased expression of Ki-67, a proliferation marker (**Figures 1C, D**).

The expression levels of XIAP and survivin in SMAC-KO were highly decreased (**Figure 1E**). Survivin, also known as baculoviral inhibitor of apoptosis repeat-containing 5 (BIRC5), is a member of the IAP family. It binds to SMAC/Diablo and prevents caspase activation, thereby leading to negative regulation of apoptosis with inhibition of this interaction promoting apoptosis (23).

We also assessed the effect of SMAC-KO on cell migration by comparing control and SMAC-KO A549 cells using a wound-healing assay (**Figures 1F, G**). A fixed-width scratch was created in a cell monolayer, and the progress of the cell migrating front was monitored using a digital camera coupled with a microscope. In comparison to control cells, which closed 81% of the gap after 24 h, SMAC-KO cells showed attenuated migration, closing only 25% of the gap (**Figures 1F, G**). Finally, we analyzed whether apoptosis is activated in SMAC-KO cells (**Figure 1H**) and found no significant different in the apoptotic cells between SMAC-expressing and SMAC-KO cells.

### SMAC-KO cells produce very small tumors with inhibited proliferation and activated apoptosis

**Figure 2:**
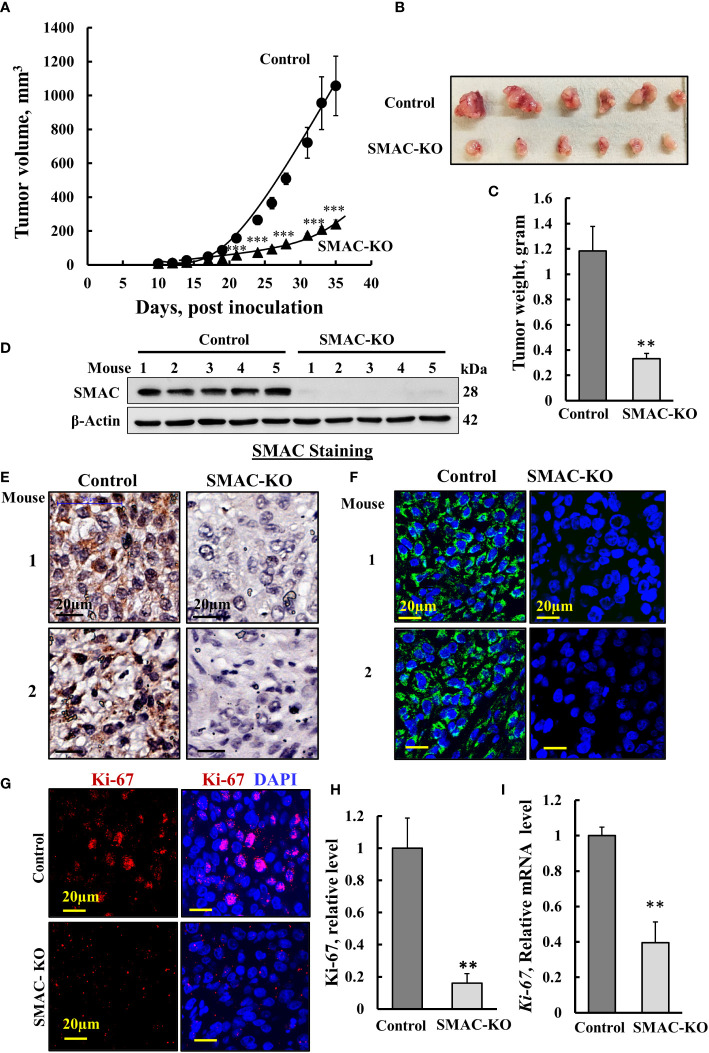
Inhibited cell proliferation in SMAC-KO A549-cell lung cancer xenograft. Control or SMAC-KO A549 cells (3x10^6^cells/mouse) were inoculated into athymic female mice (6 mice/group). Tumor volumes were monitored (using a digital caliper) for 35 days. In **(A)**, xenograft sizes were measured on the indicated days, and the calculated average tumor volumes are presented as means ± SEM, *** p > 0.001; a two tailed Student’s t-test was performed to calculate the statistical significance. Tumors from mouse A549 cell xenografts were dissected, photographed **(B)**, and weighed **(C)**. Immunoblot analysis of SMAC in protein extracts from tumors derived from control and SMAC-KO A549 cells **(D)**. Sections of paraffin-embedded control and SMAC-KO A549 cell-derived tumors were immunostained using specific antibodies for SMAC expression using IHC **(E)** or IF **(F)** and for the nuclear proliferation marker Ki-67 using IF **(G)** and its quantification **(H)**. q-RT-PCR analysis of Ki-67 mRNA **(I)**. Results represent the means ± SEM (n = 3) **p ≤ 0.01; ***p ≤ 0.001.

Next, we tested whether SMAC-KO A549 cells could form tumors in nude mice, with tumor growth followed for about 35 days. While the tumor volume of control A549 cells grew exponentially and increased over 22-fold to a volume of 1110 mm^3^, the SMAC-KO cell-derived tumor increased to a volume of 250 mm^3^, 78% smaller than the tumors in the control cells (**Figures 2A, B**), with a 70% decrease in weight (**Figure 2C**). Immunoblot, IHC, and IF analyses of the tumors indicated, as expected, that SMAC was not expressed in the tumors derived from SMAC-KO cells (**Figures 2D–F**). The inhibited cell proliferation in the SMAC-KO-derived tumors was reflected in an 80% decrease in the expression level of the cell proliferation factor, KI-67, as shown by IF staining (**Figures 2G, H**) and qRT-PCR analysis (**Figure 2I**).

**Figure 3:**
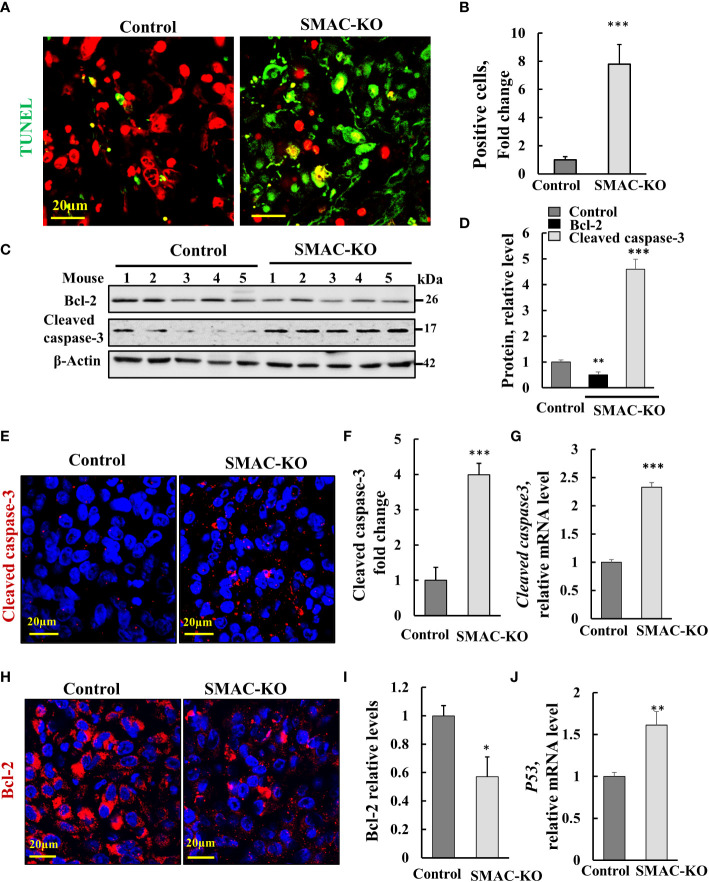
SMAC-KO-derived tumors showed apoptotic cell death. Sections of paraffin-embedded control and SMAC-KO A549 cell-derived tumors were stained with TUNEL with propidium iodide used as counter stain **(A)**, and quantified **(B)**. Immunoblot analysis of Bcl-2 and cleaved caspase-3 in protein extracts from tumors derived from control and SMAC-KO A549 cells **(C)** and their quantification **(D)**. Sections were also IF stained for cleaved caspases-3 (E,F) or Bcl-2 **(H, I)**. Cleaved caspase-3 **(G)** and p53 **(J)** mRNA levels were also analyzed by q-RT-PCR. Results represent the means ± SEM (n = 3) *p ≤ 0.05, **p ≤ 0.01; ***p ≤ 0.001.

The marked inhibition of tumor growth in the SMAC-KO-cell xenograft resulted from inhibition of cell proliferation, as reflected in the highly reduced Ki-67 expression (**Figures 2G–I**). However, it may also be the result of cell death activation. Accordingly, apoptotic cells were analyzed *in situ*, by TUNEL staining of tumor sections derived from control and SMAC-KO cells (**Figures 3A, B**). While only a few TUNEL-positive cells were apparent in the control tumors, the majority of the cells in the SMAC-KO tumors were TUNEL-positive, with staining co-localized with propidium iodide (PI) nucleus staining (**Figures 3A, B**).

Activated apoptosis is also shown by the increased levels of cleaved/activated caspase-3, analyzed by immunoblotting (**Figures 3C, D**) and IF staining (**Figures 3E, F**) and q-RT-PCR (**Figure 3G**.) In addition, the expression levels of the anti-apoptotic protein Bcl-2, as assayed using IF staining, and Finally, the expression levels of the multifaceted function, tumor suppressor p53 increased (**Figure 3J**). The results suggest that apoptosis is activated in tumors derived from cells lacking SMAC.

### SMAC-depletion altered tumor morphology and the microenvironment

**Figure 4:**
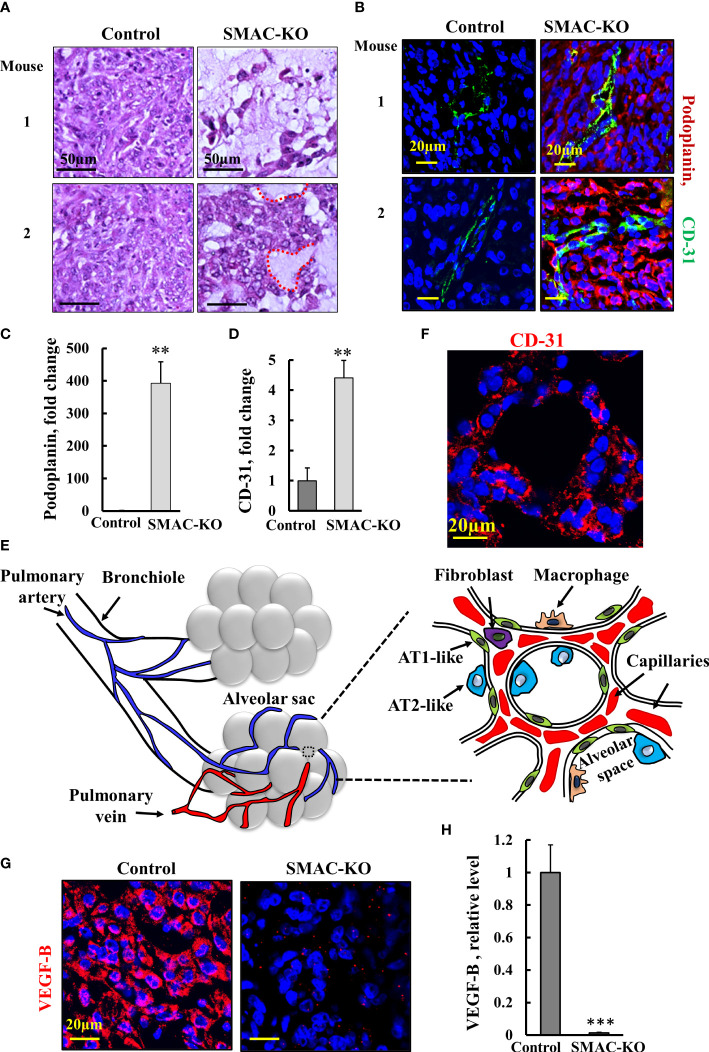
Morphology alterations in SMAC-KO-derived tumors. Representative sections from control and SMAC-KO A549 cell-derived tumors stained with H&E with cell-free areas circled **(A)**. Representative IF staining and quantification of sections of control and SMAC-KO A549 cell-derived tumors stained with specific antibodies against podoplanin **(B, C)** or CD-31 **(B-D, F)**. A schematic presentation of the morphological changes in the SMAC-KO tumors showing reorganization into alveoli-like structures with organized blood capillary and AT2-and AT1-like cells **(E)**. IF staining control and SMAC-KO A549 cell-derived tumors for VEGF-B **(G)** and its quantification **(H)**. Results represent the means ± SEM (n = 3) **p ≤ 0.01, ***p ≤ 0.001.

Hematoxylin and eosin (H&E) staining of sections from control- and SMAC-KO cell-derived tumors demonstrated that SMAC-depleted tumors showed a different morphology with cell-free areas that may resemble alveolar-like clusters of lung tissue (**Figure 4A**). As the tumor is derived from A549 cells, considered as alveolar epithelial type II (AT2) cells (24), it can trans-differentiate into alveolar epithelial type I (AT1) (25). We analyzed the expression of the AT1 cell marker podoplanin, a membranal mucin-type sialoglycoprotein (25). A massive increase (400-fold) in the podoplanin expression level was observed in the SMAC-KO tumors (**Figures 4B, C**). This may suggest that the AT2 A549 cells in the SMAC-KO-derived tumors had undergone differentiation into AT1-like cells.

Staining for the endothelial cell marker CD-31, as present in blood vessels, revealed over 4-fold higher staining in the SMAC-KO tumors relative to the SMAC-expressing tumors (**Figures 4B-D**). Importantly, the cells positively stained for CD-31 created a chain around the podoplanin-expressing cells. The CD-31-positive cell organization seems to closely resemble the normal physiological alveolar endothelial arrangement and not tumor angiogenesis.

Analysis of the pulmonary-associated surfactant proteins (SFTP-C) C expressed by AT2 cells in the SMAC-expressing and depleted tumors, showed non-homogenous expression of SETP-B, exhibiting low, medium, and high levels of SFTP-C (**Figure S1**). Generally, there was no major difference in the expression levels of SFTP-C between SMAC-expressing and -depleted tumors, although the percentage of the three sub-groups was slightly changed.

The changes in SMAC-KO tumor morphology, along with the differentiation of AT2-like cells into AT1-like cells and the blood capillary organization, suggest the formation of glandular/alveoli-like structures (**Figure 4E, F**).

Tumors often express angiogenic factors at high levels to induce neovascularization (26). Among all known angiogenic factors, vascular endothelial growth factor B (VEGF-B), *via* binding to its receptor (VEGFR), a tyrosine kinase receptor, modulates angiogenesis, vascular permeability, vessel survival, and vascular remodeling. As found for many cancers, VEGF was highly expressed in SMAC-expressing tumors, but its levels were dramatically decreased by about 100-fold in the SMAC-KO-derived tumors (**Figures 4G, H)**).

**Figure 5:**
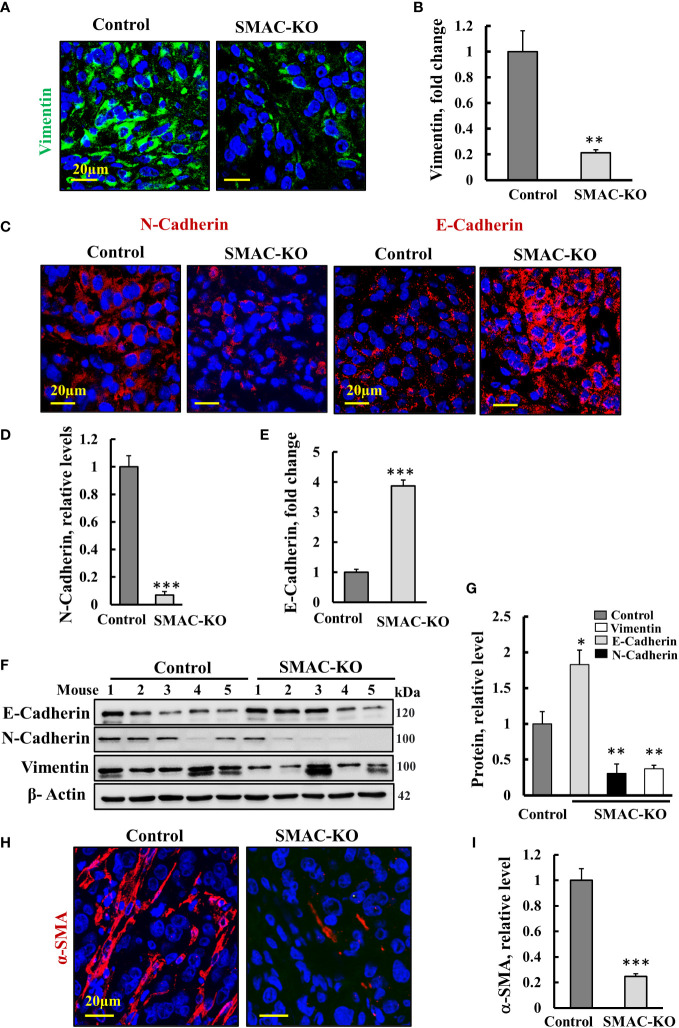
Microenvironment alterations in SMAC-KO-derived tumors. Representative IF staining and quantification of sections from control and SMAC-KO A549 cell-derived tumors stained with specific antibodies against vimentin **(A)**, N-cadherin and E-cadherin **(C)** and their quantification **(B, D, E)**. Immunoblotting of E-cadherin, N-cadherin, and vimentin **(F)** and their quantification **(G)**. IF staining of α-SMA and its quantification **(H, I)**. Results represent the means ± SEM (n = 3) *p ≤ 0.05, **p ≤ 0.01; ***p ≤ 0.001.

The tumor metastatic potential is attenuated by the epithelial to mesenchymal transition (EMT) (27). To follow EMT, we analyzed the expression of epithelial and mesenchymal cell markers N-cadherin (type I cell–cell adhesion glycoproteins), vimentin (intermediate filaments), and E-cadherin, an epithelial cell marker (28) (**Figures 5A–E**). As expected, for reversed EMT, IF staining showed that vimentin and N-cadherin expression levels were highly decreased, while those of E-cadherin was increased 4-fold in the SMAC-KO tumors relative to their expression in the control tumors (**Figures 5A–E**). Similar results were obtained by immunoblotting (**Figures 5F, G**).

Finally, IF-staining of SMAC-expressing tumors for the alpha smooth muscle actin (α-SMA) showed strong staining of cells, exhibiting the long, spindle-shaped morphology characteristic of fibroblasts. This staining was decreased by about 70% in the SMAC-KO tumors (**Figures 5H, I**). The results clearly show that SMAC-depleted tumors underwent morphological and microenvironment modulation.

### SMAC depletion reduced tumor inflammation and immunosuppression

**Figure 6:**
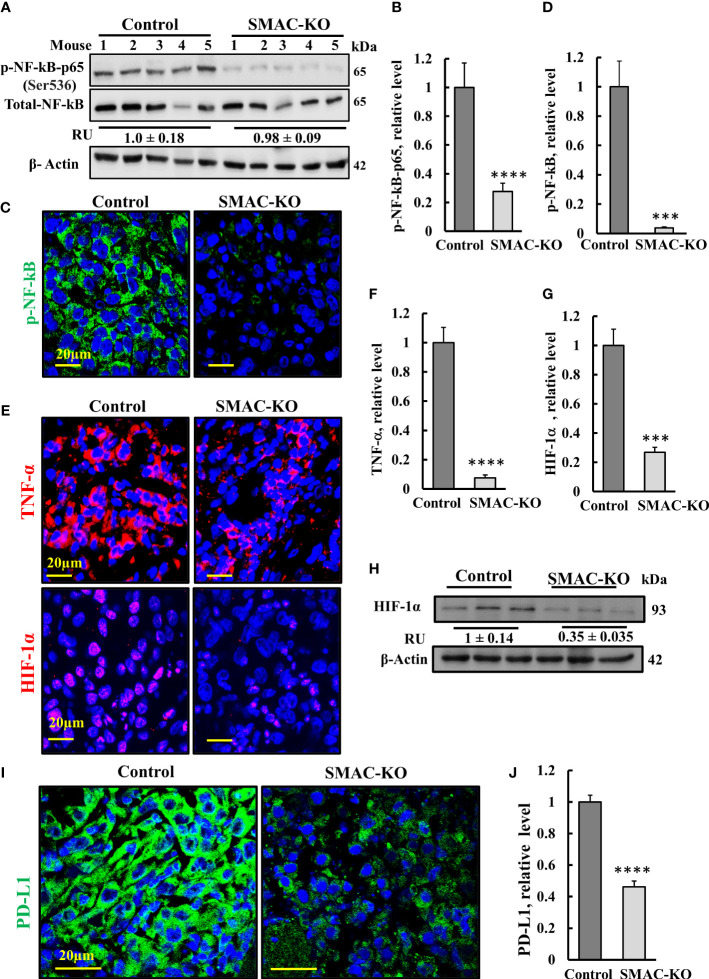
SMAC-KO-derived tumors showed altered expression of decreased inflammation and immunity-related proteins. Immunoblot of protein extracts from control and SMAC-KO A549 cell-derived tumors for NF-kB and p-NF-kB(p65) **(A)** and quantification (**B**). Average relative levels of NF-kB are presented as relative units (RUs) at the bottom of the blot. Sections of control and SMAC-KO A549 cell-derived tumors were subjected to IF staining with specific antibodies against p-NF-kB(p65) **(C)** and its quantified staining intensity **(D)**. IF staining of control and SMAC-KO tumors for TNF-α and HIF-1α **(E)** and their quantified staining intensity **(F, G)**. Immunoblotting of HIF-1α, with average relative levels presented as RUs at the bottom of the blot **(H)**. IF staining for PD-L1 **(I)** and its quantification **(J)**. Results represent the means ± SEM (n = 3) ***p ≤ 0.001. ****p ≤ 0.0001.

Inflammation is often associated with the development of cancer and promotes all stages of tumorigenesis [24]. Cancer cells in the tumor are surrounding stromal and inflammatory cells that are engaged in well-orchestrated reciprocal interactions to form an inflammatory tumor microenvironment (TME) (29). To test whether SMAC deletion affects tumor inflammation and immunity, we analyzed the expression of several inflammation- and immunity-related proteins (**Figure 6**).

Nuclear factor kappa B (NF-κB) is a network hub that consists of homo- and heterodimers of five distinct proteins: RelA (p65), RelB, c-Rel, p105 (NF-kB1), and p100 (NF-kB2) (30, 31), It coordinates many signals that drive proliferation, inflammation, oncogenesis (32), and innate immunity (33). The expression levels of the phosphorylated NF-κB/RelA (p65) and (p-NF-κB) in the SMAC-KO tumors were highly reduced by 70% (immunoblotting), and IF analysis showed an over 90% decrease relative to its levels in SMAC-expressing tumors (**Figures 6A–D**).

The decrease in p-NF-κB levels in SMAC-KO tumors may also be associated with an increased expression level of p53 (**Figure 3J**) with its transcriptional antagonism with NF-kB (34, 35). NF-κB regulates the expression of many inflammatory cytokines including tumor necrosis factor-α TNF-α. The results indicate that the levels of TNF-α were high in the control mice, but reduced in the SMAC-KO tumors (**Figures 6E, F**).

The transcription factor HIF-1α orchestrates the expression of a vast number of essential cellular functions in genes, affecting cancer progression associated with angiogenesis (as VEGF), metabolism (hexokinase, glucose transporters), cell survival, and proliferation (TGF-*α*, C-Myc). In SMAC-KO tumors, the levels of HIF-1α, as revealed by IF and immunoblotting, were reduced by about 70%, relative to its levels in SMAC-expressing tumors (**Figures 6E, G, H**).

Finally, we compared the expression of programmed cell death ligand 1 (PD-L1) in control- and SMAC-KO cell-derived tumors (**Figures 6I, J**). IF staining of PD-L1 using specific antibodies demonstrated a decrease of about 50% in PD-L1 expression levels in the SMAC-KO cell-derived tumors. PD-L1 interaction with its receptor, PD-1, compromises T-cell-mediated immune surveillance, promoting cancer cell progression. Thus, the decreased expression of PD-L1 in SMAC-lacking tumors was expected to decrease immunosuppression of the cancer.

The overall results suggest that SMAC is required for tumor-associated inflammation and immunity, and reduction in PD-L1 in its absence can advance immunotherapeutic strategies.

### Mass spectrometry analysis of the differentially expressed protein in SMAC-KO cells

**Figure 7:**
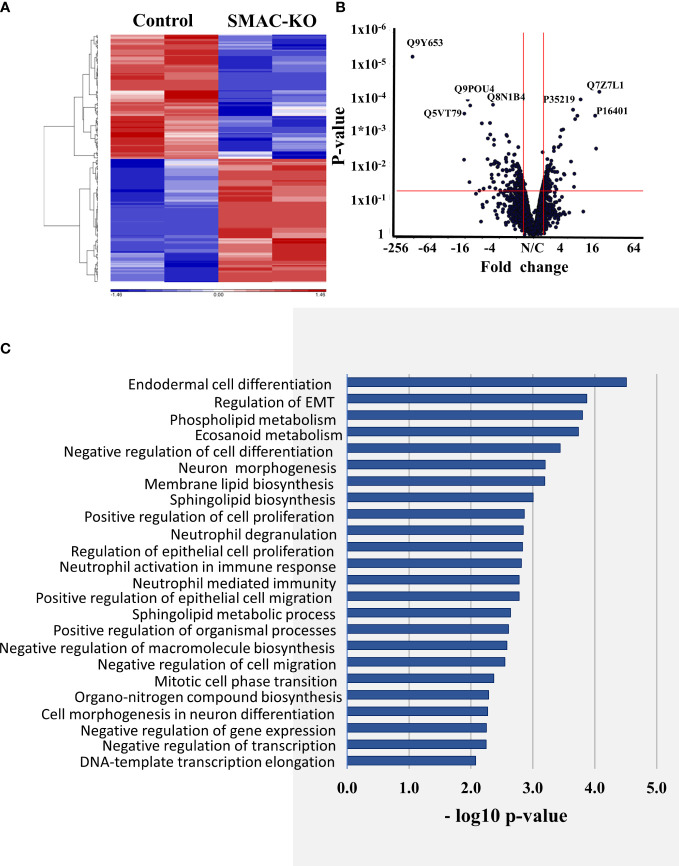
Differentially expressed proteins in SMAC-KO- and SMAC-expressing cells. Human proteins differentially expressed between SMAC-expressing and SMAC-depleted A549 cells, as analyzed by LC-HR-MS/MS. Human proteins presenting at least one unique peptide, those with a p-value of 0.05 and linear fold change of +/- 1.6 are presented. **(A)** Hierarchical clustering of the 231 proteins found to be differentially expressed between SMAC-expressing and SMAC-depleted cells. The color scale of the standardized expression values is shown. **(B)** Volcano plots showing p-values as a function of fold change in SMAC-KO- relative to SMAC-expressing cells. Significantly enriched functional groups in the proteins showing changed expression based on the David Gene Ontology system **(C)**.

**Figure 8:**
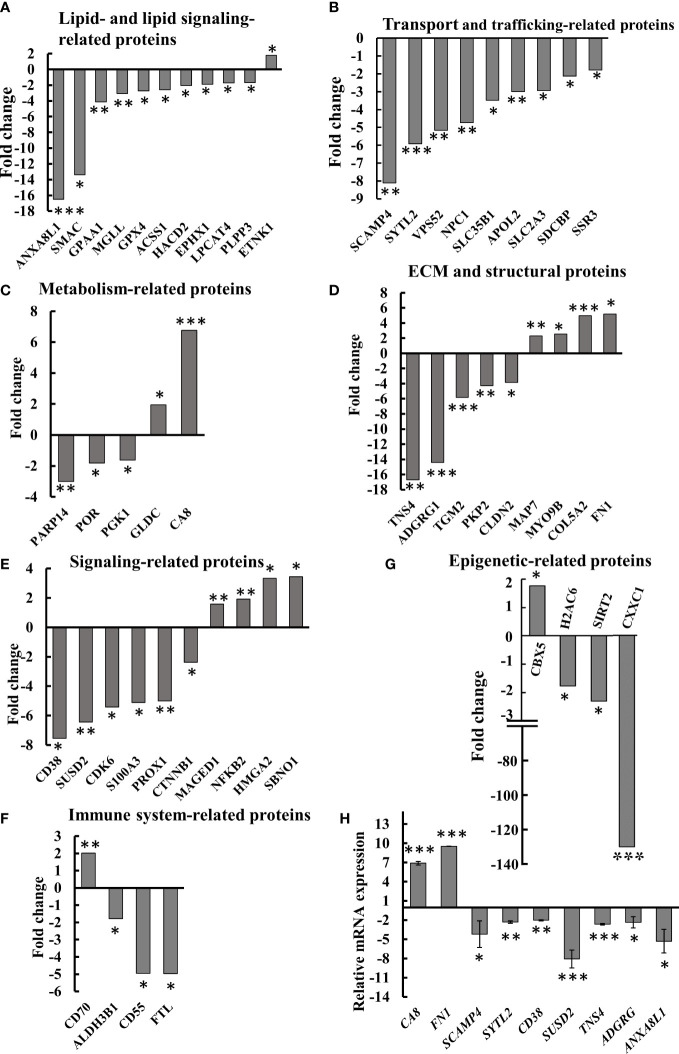
Differential expression of lipid-, metabolism-, transport- and trafficking-, ECM- and signaling-related proteins in SMAC-KO A549 cells. Quantitative analysis of the LC-HR MS/MS data. Differentially expressed lipid-related proteins **(A)** transport- and trafficking-related proteins **(B)** metabolism-related proteins **(C)** ECM and structural proteins **(D)**, cell signaling related proteins **(E)**, immune-response-related proteins **(F)** and epigenetics-related proteins **(G)** are presented in terms of fold change of expression in SMAC-KO A549, relative to A549 cells. q-RT-PCR analysis of mRNA levels of selected proteins **(H)**. *p ≤ 0.05, **p ≤ 0.001, ***p ≤ 0.0001.

To identify proteins showing different expression levels in A549 cells depleted of SMAC, and their association with inhibited cancer cell proliferation and altered tumor TME, inflammation, and immunity, CRISPR/Cas9 SMAC/Diablo-depleted cells and cells expressing SMAC were subjected to LC-HR MS/MS, proteomics, and functional enrichment analyses (**Figures 7**, **8**).

After filtering for human proteins, which had at least one unique peptide, about 4,636 proteins were submitted for subsequent analysis. Of the differentially expressed proteins (p-value < 0.05 and fold change |FC| > 1.6) between cells expressing and depleted of SMAC, 115 proteins were upregulated and 116 were downregulated. As expected, SMAC/Diablo was detected in the control cells, but not in the SMAC-KO cells (**Table S3**).

The hierarchical clustering of the differently expressed proteins in cells expressing and depleted of SMAC (**Figure 7A**) and the volcano plots (**Figure 7B**) showed that larger numbers of the differentially expressed proteins were down- or upregulated.

An enrichment analysis of the proteins differentially expressed between cells expressing and depleted of SMAC was performed using the Gene Ontology (GO) databases for biological processes (**Figure 7C**) (36, 37). The proteins with altered expression upon SMAC depletion were sub-grouped according to cellular functions. These included proteins associated with lipids and lipid-signaling molecules (**Table S3**, **Figure 8A**), transport and intracellular trafficking (**Table S4**, **Figure 8B**), metabolism (**Table S5**, **Figure 8C**), extracellular matrix (ECM) and structural proteins (**Table S6**, **Figure 8D**), cellular signaling (**Table S7**, **Figure 8E**), immune response including neutrophil-mediated immunity and neutrophil degradation (**Table S8**, **Figure 8F**), DNA- and RNA-associated processes (**Table S9**), protein synthesis and degradation (**Table S10**), and epigenetics (**Table S11**, **Figure 8G**).

Selected proteins from the various groups are presented in **Figure 8A**. The lipid and lipid-signaling molecules (**Table S3**, **Figure 7A**) included increased expression of ethanolamine kinase 1 (ETNK1), mediating ethanolamine phosphorylation, a rate-controlling step in PE biosynthesis (38). All other proteins in this group were downregulated (**Table S3**, **Figure 8A**). These included: phospholipid phosphatase 3 (PLPP3) and 2, a phospholipid phosphatase that catalyzes the conversion of phosphatidic acid (PA) to diacylglycerol (DG); mono-glyceride lipase (MGLL), which converts mono-acylglycerides to free fatty acids and glycerol; and glycosylphosphatidylinositol anchor attachment 1 protein (GPAA1), which is involved in the glycosylphosphatidylinositol-anchor biosynthesis pathway that is part of glycolipid biosynthesis. Annexin A8-like protein 1 (ANXA8L1) functions as an anticoagulant, and indirectly inhibits the thromboplastin-specific complex.

The expression levels of several intracellular trafficking-related proteins and transporters were mostly reduced (**Table S4**, **Figure 8B**). These included: signal sequence receptor 3 (SSR3) a subunit of the translocon-associated protein (TRAP) complex which facilitates the translocation of proteins across the ER membrane; solute carrier family 2; glucose transporter member 3 (SLC2A3); apolipoprotein L2 (APOL2), which is involved in the movement of lipids in the cytoplasm and allows the binding of lipids to organelles; solute carrier family 35 member B1 (SLC35B1) which facilitates UDP-galactose transmembrane transport; intracellular cholesterol transporter 1 (NPC1); vacuolar protein sorting-associated protein 52 homolog (VPS52), which is involved in retrograde transport from early and late endosomes to the trans-Golgi network; synaptotagmin-like protein 2 (SYTL2), a protein required for cytotoxic granule docking at the immunologic synapse; and secretory carrier-associated membrane protein 4 (SCAMP4), a membrane protein, with components of regulated secretory carriers in exocrine, neural, and endocrine cells, all are which downregulated 6–8 fold in SMAC-KO cells compared with wild-type A549 cells.

The expression of several metabolism-related proteins was altered in the SMAC-depleted cells (**Table S5**, **Figure 8C**). These include the carbonic anhydrase-related protein (CA8), a non-active isoform with the only known biochemical function is affecting IP3 binding to its receptor IP3R1 on the ER, thereby modulating Ca^2+^ signaling (39). Mitochondrial glycine dehydrogenase (GLDC), catalyzing the degradation of glycine, is also upregulated in SMAC-KO cells, while glycolytic enzyme.

Phosphoglycerate kinase 1 (PGK1), microsomal NADPH-cytochrome P450 reductase (POR), and protein mono-ADP-ribosyltransferase (PARP14), which mediates mono-ADP-ribosylation of glutamate residues on target proteins, were significantly downregulated.

Another group of proteins whose expression was modified is associated with the organization and functions of extra-cellular matrix (ECM) and structural proteins (**Table S6**, **Figure 8D**), which were found to be significantly reduced (3–17-fold), while others increased 2–6 fold. The downregulated proteins included tensin-4 (TNS4), which is involved in cell migration and links the signal transduction pathways to the cytoskeleton; and protein-glutamine gamma-glutamyltransferase 2 (TGM2), catalyzing the cross-linking of proteins between Gln and Lys residues (40). Several cell adhesion proteins such as G-protein coupled receptor G1 (ADGRG1), plakophilin-2 (PKP2), and claudin-2 (CLDN2) were also significantly reduced.

Some proteins were found to be upregulated in SMAK-KO cells, such as fibronectin (FN1) which is involved in cell adhesion and motility, the wound healing process, the collagen alpha-2(V) chain (COL5A2), a connective tissue component that binds to DNA, heparan sulfate, heparin, thrombospondin, and insulin. Unconventional myosin-IXb (MYO9B) and microtubule-stabilizing protein ensconsin (MAP7) expression levels were also increased upon SMAC depletion (**Figure 8D**, **Table S6**).

The proteomics data explored proteins related to signaling pathways, development, and differentiation whose expression levels were altered in SMAC-KO cells (**Table S7**, **Figure 8E**). ADP-ribosyl cyclase/cyclic ADP-ribose hydrolase 1 (CD38) synthesizes the second messenger cyclic ADP-ribose and nicotinate-adenine dinucleotide phosphate decreasing it by about 8-fold. Protein strawberry notch homolog 1 (SBNO1) and high mobility group protein (HMGI-C) were increased over 3-fold. The p100 subunit of NF-kB, a master transcription factor, and the melanoma-associated antigen D1 (MAGED1), a cell cycle inhibitor and inducer of NGFR-mediated apoptosis were also upregulated. Other proteins such as catenin beta-1 (CTNNB1), a key downstream component of the canonical Wnt signaling pathway; cyclin-dependent kinase 6 (CDK6) which functions as a cell cycle and differentiation regulator; and sushi domain-containing protein 2 (SUSD2) that negatively regulates cell cycle G1/S phase transition were decreased 3–7-fold. SMAC-KO also altered the expression of immune response-related proteins, including neutrophil-mediated immunity (**Table S8**, **Figure 8F**).

Among the proteins whose expression levels were decreased upon SMAC depletion was ferritin light chain (FTL), a subunit of ferritin that is the main form of iron storage protein and is known to influence tumor immunity (41), which decreased over 5-fold. Similarly, CD55 decreased about 5-fold when overexpressed in tumors, resulting in immune escape adopted to avoid recognition by the immune system or of survival from antibody-mediated immunotherapy (42) (**Fig. 8F**, **Table S8**). CTSD (cathepsin D), a lysosomal aspartic, has been related to immune response and regulation of programmed cell death. The aldehyde dehydrogenase 3 family, member B1 ALDH3B1 is proposed to play a significant role in the tumor immune landscape by modulating immunocytes (43) **Table S8**.

The expression of proteins related to DNA and RNA (**Table S9**) also was modified in SMAC-KO cells as schlafen family member 11 (SLFN11), an inhibitor of DNA replication, was increased about 14-fold, while the expression levels of protein bicaudal C homolog 1, an ARNA binding protein that acts as a negative regulator of Wnt signaling and is upregulated in oral cancer tissues, was decreased over 2-fold.

Finally, expression of proteins associated with epigenetics (**Table S11**, **Figure 8G**) was also modified as Histone H1.5, a regulator of individual gene transcription and overexpression in prostate cancer, increased about 12-fold, while the expression of CXXC-type zinc finger protein 1, a transcriptional activator was reduced by 135-fold in SMAC-KO cells.

Alterations in the expression of selected proteins at the mRNA level were analyzed using q- RT-PCR (**Figure 8H**). The obtained results validated the proteomic results.

These results point to the involvement of SMAC in many cell-signaling pathways and activities, as mediated *via* its interaction and modulation of key proteins in cellular pathways.

## Discussion

Mice lacking the pro-apoptotic protein SMAC/Diablo are viable, and grow and mature normally, without any histological abnormalities, and exhibit responses to all types of apoptotic stimuli such as SMAC-expressing mice, suggesting that this protein is not essential for apoptosis (13). Interestingly, we showed that in spite of being a pro-apoptotic protein, SMAC is overexpressed in many cancer types (19), suggesting that it possesses an additional non-apoptotic function that is important in tumor development.

Indeed, we demonstrated that SMAC depletion in cancer cells using specific si-RNA led to multiple effects, including reduced cell proliferation and decreased phospholipid levels, and in tumor silencing, SMAC expression inhibited tumor growth, and the residual “tumor” showed cell differentiation and reorganization to alveoli-like structures (19, 20). Moreover, CRISPR/Cas9 SMAC depleted cells showed inhibited proliferation of cancer cells, but not non-cancerous cells (20). This further points to the importance of SMAC for the cancer cell. Here, using CRISPR/Cas9 SMAC-depleted cells, we further explored the non-apoptotic functions of SMAC using proteomic analysis and a lung cancer mouse model.

**Figure 9:**
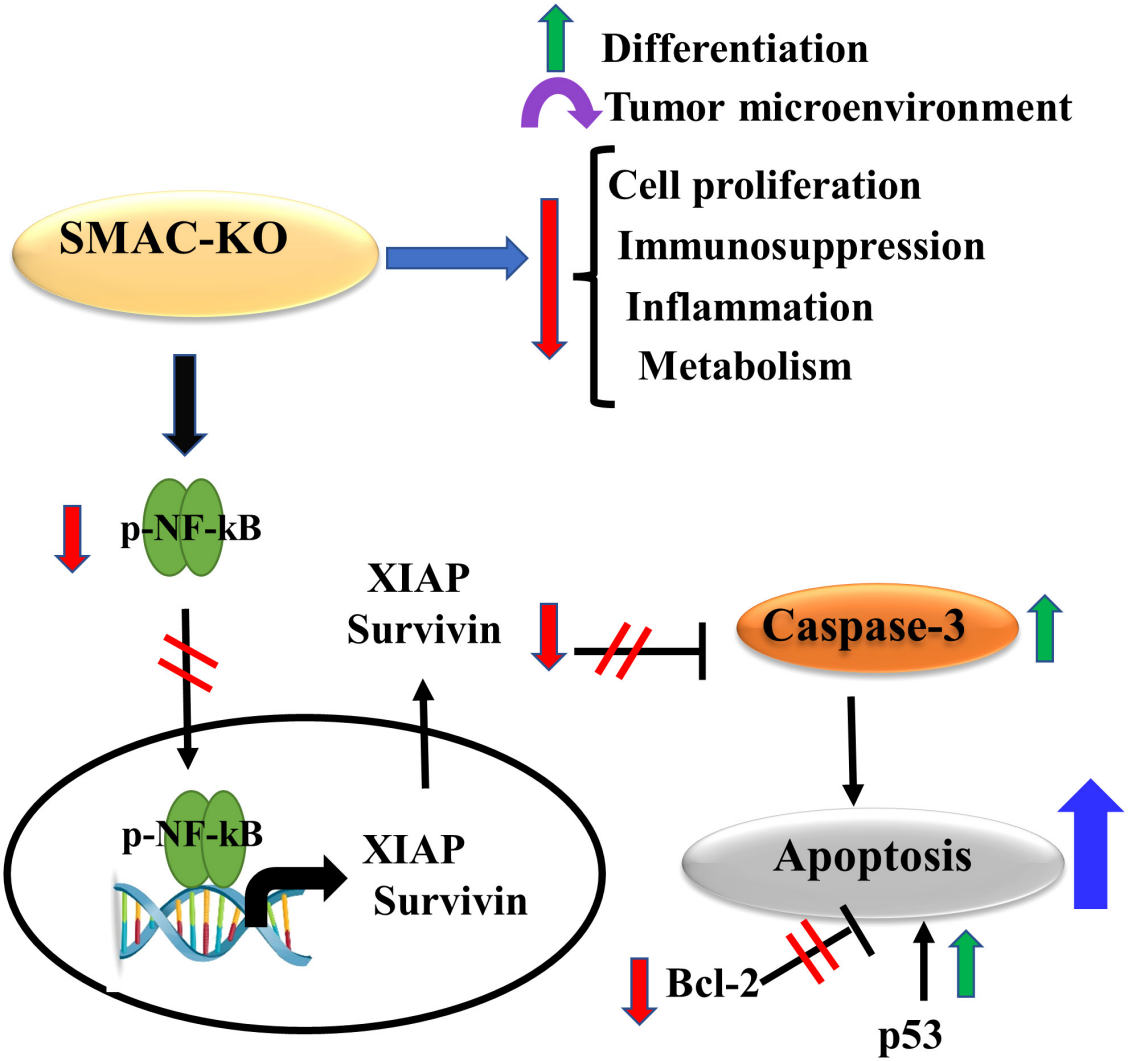
Proposed model for SMAC multifunction’s in cancer. The small tumors formed by the SMAC-KO cells showed reversal of the unique properties of the tumor. These include inhibited cell proliferation, reduced metabolism, inflammation and immunosuppression, altered TME, and induced differentiation. In addition, they lead to apoptosis induction due to several factors that include increased caspase activity and p53 expression, and reduced expression levels of Bc-l2, survivin, and XIAP.

The results show that SMAC is also involved in regulating lipid synthesis, cell proliferation, the TME, inflammation, and immunity. The involvement of SMAC in these processes is summarized in **Figure 9**, suggesting that targeting SMAC would result in attacking many cancer properties.

SMAC, as a pro-apoptotic protein that interacts and antagonizes IAPs activity, allows the activation of caspases and apoptosis. SMAC-derived peptides, peptidomimetics, to target IAPs and induce apoptosis were produced and applied as cancer therapy (44–48). However, SMAC-mimetic treatment increases resistance to DNA-damaging chemotherapeutic agents rather than reducing it (49). In addition, IAP antagonists have been shown to interact with tumor necrosis factor receptor-associated factor 2 (TRAF2), shown to interact with SMAC (19, 20).The resulting complex reportedly antagonizes the activation of caspase-8, hence, inhibiting TNF receptor-mediated apoptosis (14). Moreover, SMAC-mimetics clinical trials for hematological and solid cancers showed variable and limited results (50). The SMAC mimetics may modulate other SMAC-interacting protein activities reflected in the SMAC non-apoptotic activities and may explain their lack of effectiveness on tumor growth. Thus, they are not approved for clinical treatment of cancer (50).

It should be noted that, in this study, we used A549 lung cancer-derived xenografts that were developed in nude mice, and that the tumors were analyzed for microenvironment, inflammation and immunity. Therefore, the properties of these mice should be considered. Nude mice carry a FOXN1 mutation that leads to athymic phenotype lacking αβ-T cells, Th1, Th2, Th17, and Treg cells, but they also have cells of myeloid origin, such as macrophages, granulocytes, antigen presenting cells (APCs), natural killer (NK) cells, B cells and T cells, such as NK T and γδ-T cells (51).

These nude mice, however, were used in various studies related to the TME including in A549 lung cancer- or MDA-MB231 breast cancer-derived tumors, as well as other cancer types which showed that, in these mice, the TME including tumor-associated fibroblasts, immune cells, and cellular components (e.g., cytokines, chemokines, growth factors) are present in the TME and can be modulated by various treatments (52, 53). In addition, these mice were used in several studies using A549 cells xenograft and analyzed PD-L1 expression (54–56).

### SMAC depletion inhibited tumor growth and induced apoptosis

SMAC-depleted A549 lung cancer-derived xenografts in mice showed a low capacity to develop tumors, as expected from their reduced proliferation (**Figure 2G**). However, the inhibition of tumor growth could also be due to activation of apoptosis. Interestingly, while SMAC/-KO cells in culture, showed no significant apoptosis (**Figure 1G**), the SMA-KO-derived tumors showed massive apoptosis, as revealed using TUNEL staining, increased activated caspase-3, and reduced expression of the anti-apoptotic protein Bcl-2, XIAP and increased p53 (**Figure 3**).

Moreover, survivin/BIRC5, overexpressed in cancers and a prognostic marker of several cancers (57), was highly reduced in the SMAC-KO cells (**Figure 1**). As survivin/BIRC5 binds to SMAC/Diablo and prevents caspase activation thereby leading to negative regulation of apoptosis (23), its decreased levels would promote apoptosis. Survivin/BIRC5 silencing leads to apoptosis *via* activation of p53, which was found to increase in SMAC-KO cells (58).

Survivin, however, is functionally important not only for apoptosis, but also in mitochondrial metabolism, mitosis, autophagy, promotion of cell proliferation regulation of cell division, and cell survival (59). Thus, its reduction in SMAC-lacking cells impacts not only in apoptosis regulating, but also multi-tasking, and it is an onco-therapeutic target (60).

This apoptosis may also be associated with p-NF-kB/p65. Our results show very high expression levels of p-NF-kB/p65 in tumors derived from SMAC expressing A549 cells that were highly reduced in tumors lacking SMAC. This decrease in p-NF-kB/p65 can induce apoptosis as found in other studies where apoptosis was induced when NF-kB activation was inhibited by different means (61, 62).

### SMAC depletion induced morphological changes and cell differentiation

The non-apoptotic function of SMAC is reflected in the spectrum of proteins whose expression levels were altered in the SMAC-KO cells. This includes proteins related to lipids and lipid-signaling molecules, metabolism, DNA- and RNA-associated processes, transport and intracellular trafficking, cellular signaling, ECM and structural proteins, epigenetics, protein synthesis and degradation, and immune response (**Tables S3–S11**, **Figure 8**). The proteins with altered expression in the SMAC-KO cells, presented in detail in the Results section, show a network of key regulators of cell function whose altered expression can lead to cancer cell differentiation.

Here, we focus on the morphological changes and the differentiation observed in the SMAC-KO tumors. Moreover, the inoculated A549 cells, considered as alveolar epithelial type II (AT2)-type cells (19) are considered to be not fully differentiated alveolar epithelial type II (AT2) cells (24). AT2 cells are surfactant-producing cells expressing the pulmonary-associated surfactant proteins (SFTP) A, B. Yet, in the tumors derived from SMAC-KO A549 cells, we detected AT1 cell marker podoplanin (also known as T1α or PDPN), a membranal mucin-type sialoglycoprotein, suggesting that in the absence of SMAC, AT2 cells differentiated into AT1 cells, as reported for cells in culture under certain conditions (25). PDPN is present in many types of normal cells, such as in endothelial cells in lymphatic vessels, and not only in AT1 cells (63).

The morphological changes found in the SMAC-KO cell-derived tumors resembled alveolar-like clusters of lung cancer tissue (**Figure 4**). This agrees with our previous study, in which we used si-RNA against SMAC, demonstrating that the residual tumors showed morphological changes, including cell differentiation and reorganization into glandular/alveoli-like structures (19, 20). The morphology reorganization into these structures was also reflected in the arrangement of the blood capillaries in the periphery of the alveolus to allow gas exchange.

### SMAC depletion altered the tumor microenvironment

Tumors lacking SMAC also showed an altered TME which included ECM, fibroblasts, inflammatory cells, and endothelial cells. All are important in promoting tumor progression.

Angiogenesis is the consequence of interactions between the tumor and its environment with many factors such as VEGF secreted by tumor cells and surrounding stroma stimulating the proliferation and survival of endothelial cells, leading to the formation of new blood vessels (64). Consequently, treatment strategies have focused on angiogenesis inhibition such as using anti-VEGF in non-small cell lung cancer (NSCLC) patients. However, it has been shown that anti-angiogenic therapy elicits malignant progression of tumors and increases local invasion and metastasis (65–67). Here, we demonstrated that, in contrast to the highly expressed VEGF in SMAC-expressing cells, almost no VEGF was found in the tumors derived from SMAC-lacking cells.

Epithelial-to-mesenchymal transition (EMT) is considered to be one of the steps involved in normal cells becoming cancerous (68). Using the markers for EMT, such as α-SMA, vimentin, E-cadherin, and N-cadherin, our results show that in SMAC-KO cell-derived tumors, the process of EMT was reversed (MET). Vimentin, a type III intermediate filament, normally expressed in mesenchymal cells, is considered a biomarker for EMT, and it is upregulated during cancer metastasis (69). Vimentin expression levels were highly reduced in SMAC-KO cell-derived tumors (**Figures 5A, B, F**). This is important for vimentin to be considered as a therapeutic target to inhibit cancer growth and spread (69). EMT hallmarks also include upregulated N-cadherin and downregulated E-cadherin. This is reversed in SMAC-depleted tumors (**Figures 5C–G**), suggesting that induction of MET thereby inhibited migration and metastasis.

The reversal of EMT is also reflected in altered expression of α-SMA produced by the cancer-associated fibroblasts (CAFs), most often associated with poor patient survival/outcome (70). Our results showed a high decrease in α-SMA levels in SMAC-depleted tumors when compared to control tumors (**Figures 5H, I**). This suggests decreased infiltration of CAFs. Tumor cells expressing α-SMA are predicted to have an invasive nature. These cells tend to metastasize and have a poorer prognosis (70).

Finally, alterations in the TME are also shown in the altered expression of ECM and in structural proteins in SMAC-KO A549 cancer cells (**Table S6**, **Figure 8D**). ECM is extremely versatile and performs many functions in addition to its structural role, taking part in most basic cell behaviors from cell proliferation, adhesion and migration to cell differentiation, cell death, and tissue remodeling (71, 72). Thus, these functions of the ECM are modified in the absence of SMAC.

As cancer cells utilize EMT to acquire the ability to migrate, resist therapeutic agents, and escape immunity, reversing EMT by depleting SMAC is a promising strategy for targeting cancer.

### SMAC is involved in inflammation and immunity

Our results show that in SMAC-KO cells, and in tumors established from these cells, the expression of many proteins involved in the regulation of tumor inflammation and immunity were altered, suggesting reduced inflammation and activating an immune response.

SMAC association with the immune system is reflected in the proteomic analysis of SMAC-expression and SMAC-KO cells, revealing that the expression levels of many proteins of the immune system were altered (**Table S8**, **Figure 8F**). SMAC-KO cells showed altered neutrophil-mediated immunity such as decreased expression, ferritin light chain (FTL), and CD55. Neutrophils play an essential role during an inflammatory response and participate in the initiation and regulation of the adaptive immune response at the inflammation site through interaction with antigen-presenting cells and lymphocytes (72–76). Moreover, neutrophils play a major role in the pathogenesis of cancer, including in tumor initiation, development, and progression (77, 78). The role of neutrophils in cancer is dependent on various factors and may result in a pro-tumoral or an antitumoral effect (79), with most studies indicating that neutrophils promote tumorigenesis (80, 81).

NF-κB coordinates hundreds of gene expressions involved in cell proliferation and apoptosis, stress responses to a variety of stimuli, and an innate immune response (30). Our results show very high expression levels of the activated p-NF-kB/p65 in tumors derived from SMAC-expressing A549 cells, that was highly reduced (over 90%) in SMAC-lacking tumors. This decrease in p-NF-kB/p65 led to reduced inflammation (**Figure 6**) and apoptosis (**Figure 3**), as also obtained when NF-kB activation was induced by different means (61, 62).

Pro-inflammatory NF-κB signaling is activated by at least three pathways (82). Among them is TNF-α, a pro-inflammatory cytokine that results in activating p65 that regulates inflammatory responses (83). TNF-α, acting *via* specific cell-surface receptors, can induce both apoptosis and inflammation, and can modulate the innate and adaptive immune system (84). Our results show deceased expression of TNF-α in SMAC-lacking tumors (**Figure 6**). Thus, the decreased inflammation and induced apoptosis in these tumors suggests that in cancer, SMAC is involved in TNF-α/NF-kB signaling.

In this respect, as SMAC binds IAPs, which were shown to also regulate the activation of NF-κB, and, thereby, inflammation, immunity, cell migration and cell survival, the absence of SMAC and the decrease in XIP and survivn may affect these IAP activities (10, 85).

Another player in inflammation and immunity is transcription factor HIF-1α. It influences many aspects of innate immune cells and regulates M1 macrophage polarization, dendritic-cell maturation and migration, and neutrophil NET formation and survival, and modulates various EMT transcription factors (86) [64]. HIF-1α expression level was highly reduced in SMAC-lacking tumors (**Figures 6E, G, H**), and, as discussed above, EMT and the neutrophil-associated inflammatory response and regulation of adaptive immune response regulation were altered (**Table S8**, **Figure 8F**).

Different types of cancers develop immune escape mechanisms such as the expression of high levels of PD-L1 protein. PD-L1 and its receptor PD-1 are two typical immune checkpoints (ICs) whose interaction concedes T-cell-mediated immune surveillance, thus, promoting cancer cell progression (87). The binding of PD-L1 to PD-1 inhibits T-cell proliferation and activity, leading to tumor immunosuppression (88). Accordingly, cancer immunotherapy, with IC inhibitors (ICIs), such as antibodies against PD-1 or PD-L1 to block PD-L1 or PD-1 on activated T cell membranes, were developed (89, 90), with ICIs significantly enhancing antitumor immunity and prolonging survival (91). However, response rates of patients are less than 40% (92), and they develop adaptive resistance and treatment toxicity (93). NSCLC is one of several tumors in which there are significant atypically upregulated expression levels of PD-L1 and PD-L2, yet the response to immunotherapy of NSCLC patients is about 30% (94).

Our results demonstrate that SMAC-KO tumors express reduced PD-L1 levels (**Figures 6I, J**). ICIs such as PD-L1 or PD-1 monoclonal antibodies have been used for cancer treatment, including for melanoma, non-small-cell lung cancer, gastric cancer, and breast cancer (95).

The expression of PD-L1 in cancer cells is regulated by multiple signaling pathways that include NF-κB, (96), which induce PD-L1 gene expression, which is abolished by NF-κB inhibitors (97, 98). It has also been reported that TNF-α upregulates PD-L1 expression in cancer cells (99). Thus, the decrease in p-NF-kB and TNF-α levels demonstrated in SMAC-depleted tumors can explain the observed decrease in PD-L1. In addition, inhibition of PD-L1 expression promotes apoptosis in cancer cells (100), in agreement with the increased apoptosis in the SMAC-lacking tumors.

The decrease in EMT in SMAC-KO tumors observed here may also be induced by the decrease in PD-L1 shown to promote EMT (101, 102). It should be noted that chemotherapy or radiation could decrease the response rates to the PD-L1/PD-1 blockade by increasing PD-L1 expression in cancer cells (103).

Interestingly, in a previous study it was shown that expression of a lentiviral vector encoding the cytosolic form of SMAC (tSMAC) elicits proinflammatory cell death that is sufficient to activate adaptive anti-tumor immune responses in cancer (12). Moreover, the infiltration of effector T cells within tumors treated with tSMAC show low expression of PD-1 (12). It was also shown that SMAC mimetics, which competitively inhibit SMAC-cIAP-1/2 interaction and thus repress anti-apoptotic functions of IAP proteins, elicit proinflammatory cell death in cancer cells that engages an adaptive antitumor immune response (104). These finding support our results proposing an additional function for SMAC related to anti-tumor immunity. Taken together, our finding that SMAC depletion significantly reduced the level of PD-L1, suggests that the combination of si-RNA against SMAC with ICIs may be a more effective treatment for cancer than ICIs alone. Moreover, si-SMAC also inhibits cell proliferation, modulates EMT, promotes tumor tissue reorganization, and increases immunogenicity; thus, its combination with anti-PD-L1 therapy could effectively treat tumors.

In summary, for the first time, we demonstrated that, in cancer, SMAC/Diablo possesses several functions distinguished from its well-known activation apoptosis *via* binding the XIAP. Proteomics analysis of SMAC-KO cells revealed that the absence of SMAC resulted in altered expression of proteins associated with a verity of cell functions from lipid transport and intracellular trafficking to metabolism, DNA- and RNA-associated processes, cellular signaling, and immunity. Moreover, SMAC-lacking tumors showed inhibited proliferation and altered oncogenic properties. These include reduced tumor growth and angiogenesis, microenvironment re-modulation, switching from EMT to MET, reduced inflammation, and inducing cell differentiation/arrangement to alveoli-like structures. In addition, the expression of PD-L1, TNF-α, and NF-kB were reduced, resulting in suppressed inflammation, enhanced immunogenicity, and apoptosis promotion.

These results suggest that in cancer cells, SMAC is involved in multiple processes that are essential for tumor growth and progression. Thus, SMAC should be considered as a potential target for the development of new approaches to treat cancer.

## Data availability statement

The original contributions presented in the study are included in the article/**Supplementary Material**. Further inquiries can be directed to the corresponding author.

## Ethics statement

All experiments were approved by the Animal Care and Use Committee of Ben-Gurion University of the Negev, as required by Israeli legislation, and all efforts were taken to minimize animal suffering.

## Author contributions

Methodology: SP and AS-K; visualization: VS-B, SP, and AS-K; formal analysis: VC-C; conceptualization: VS-B; writing - review and editing: VS-B; supervision: VS-B. All authors contributed to the article and approved the submitted version.

## Funding

This research was supported by a grant from the National Institute for Biotechnology in the Negev (NIBN).

## Conflict of interest

The authors declare that the research was conducted in the absence of any commercial or financial relationships that could be construed as a potential conflict of interest.

## Publisher’s note

All claims expressed in this article are solely those of the authors and do not necessarily represent those of their affiliated organizations, or those of the publisher, the editors and the reviewers. Any product that may be evaluated in this article, or claim that may be made by its manufacturer, is not guaranteed or endorsed by the publisher.
