# Supplementary material for: Non-apoptotic activity of the mitochondrial protein SMAC/Diablo in lung cancer: Novel target to disrupt survival, inflammation, and immunosuppression
Source: Front Oncol. 2022 Sep 14;12:992260. doi: 10.3389/fonc.2022.992260 (PMC9515501; doi:10.3389/fonc.2022.992260)
Supplement: Supplementary file 1 [file DataSheet_1.pdf]

Supplementary Data

Materials and Methods

Materials

Transfection agent JetPRIME was procured from PolyPlus transfection (Illkirch, France). Trypan blue, Triton X-100, Tween-20, hematoxylin, eosin, 4′,6-diamidino-2-phenylindole (DAPI), and dimethyl sulfoxide (DMSO) were obtained from Sigma-Aldrich (St. Louis, Missouri, USA). Dulbecco's modified Eagle's medium (DMEM) and phosphate buffered saline (PBS) were obtained from Gibco-Thermo Fisher Scientific (Waltham, MA, USA). Normal goat serum (NGS), DEPC-treated water, fetal bovine serum (FBS), trypsin, EDTA, a chemiluminescence detection kit for HRP (EZ-ECL), and penicillin and streptomycin were obtained from Biological Industries (Beit Haemek, Israel). A protease inhibitor cocktail set III, EDTA-free, was purchased from Millipore (Burlington, MA, USA). 3,3-Diaminobenzidine (DAB) was obtained from Vector laboratories (Newark, CA, USA). A TUNEL assay kit was obtained from Promega (Madison, WI, USA) Primary and secondary antibodies, their source, and their dilutions are detailed in Table S1.

Protein extraction and immunoblot

Cells were harvested and washed twice with ice-cold PBS, and the pellets were lysed on ice for 30 min in a lysis buffer (50 mM Tris-HCl, pH 7.5, 150 mM NaCl, 1 mM EDTA, 1.5 mM $MgCl_2$, 10% glycerol, 1% Triton X-100), freshly supplemented with a protease inhibitor cocktail (Calbiochem), incubated on ice for 20 min, and centrifuged (10 min, 12,000 g). The protein concentration of the supernatant was determined, and cells were stored at -80°C until used for gel electrophoresis and immunoblotting. Protein samples (10–20 μg) were subjected to SDS-PAGE and immunoblotting, using the selected primary antibodies, followed by incubation with HRP-conjugated secondary antibodies. HRP activity was determined using an enhanced chemiluminescent substrate (Pierce Chemical, Rockford, IL). Band intensity was quantified using ImageJ.

In-vitro migration and wound-healing assay

The migration of A549 cells and SMAC-KO cells was assayed using the wound-healing assay [1]. Cells were seeded in 24-well plates and grown to 95–100% confluence. Thereafter, a spatula was used to scratch a fixed-width band in the cell monolayer. This step was followed by a 24-h incubation with medium containing 1% FCS. Wound closure was monitored using a digital camera mounted on a microscope to follow the position of the migrating front at defined times.

Sulforhodamine B (SRB) cell proliferation assay

Cells were seeded in 96-well cell culture plates (8,000/well) and allowed to grow for 48 h. After washing with PBS, cells were fixed with 10% trichloroacetic acid, and stained with SRB for 20 min. Excess SRB was removed, and cells were washed with 1% acetic acid. SRB extraction was done using 100 mM Tris-base, and absorbance at 510 nm was determined using an Infinite M1000 plate reader (Tecan, Männedorf, Switzerland).

TUNEL assay

Fixed tumor sections in paraffin were used for the TUNEL assay employing the DeadEnd Fluorometric TUNEL system (Promega, Madison, WI) according to the manufacturer's instructions. Sections were deparaffinized, equilibrated in PBS, permeabilized with proteinase K (20 μg/ml in PBS), post-fixed in 4% paraformaldehyde, and incubated in TdT reaction mix (Promega) for 1 h at

37°C in the dark. Slides were then washed in 2x saline-sodium citrate (SSC) buffer, counter-stained with propidium iodide (1 µg/ml), and coverslipped with Vectashield mounting medium (Vector Laboratories, Burlingame, CA). Fluorescent images of apoptotic cells (green) and cell nuclei (red) were captured using a confocal microscope (Olympus 1X81).

Table S1. Antibodies used in the study. Antibodies against the specific protein, source, catalogue number, and the dilutions used in IHC, immunofluorescence (IF), and immunoblot are presented.

Antibody	Source and Cat. No.	Dilution	
		IHC/IF	WB
Mouse monoclonal anti-actin	Millipore, Billerica, MA, MAB1501	-	1:20000
Rabbit polyclonal anti-SMAC/Diablo	Abcam, Cambridge, UK, ab8115	1:500	1:2000
Human anti-PDL-1 antibody	AstraZeneca, Cambridge, UK, Durvalumab	1:100	-
Rabbit monoclonal anti-Ki67	Thermo Scientific, NY RM9106-s1	1:100	-
Rabbit monoclonal anti-survivin antibody	Abcam, Cambridge, UK, ab134170	-	1:2000
Rabbit polyclonal anti-Bcl2 antibody	Abcam, Cambridge, UK, ab196495	1:500	1:2000
Rabbit monoclonal anti-cleaved caspase-3 antibody	Cell Signaling Technology, Inc, MA, #9664	1:750	1:3000
Rabbit polyclonal anti-CD31 antibody	Abcam, Cambridge, UK, ab28364	1:750	-
Mouse monoclonal anti-podoplanin antibody	Abcam, Cambridge, UK, ab10288	1:750	-
Mouse monoclonal anti-VEGF-B antibody	Santa Cruz Biotechnology, TX, sc-65617	1:100	-
Mouse monoclonal anti-vimentin antibody	Abcam, Cambridge, UK, ab8978	1:500	1:4000
Rabbit monoclonal anti-E-cadherin antibody	Cell Signaling Technology, Inc, MA, #3195	1:500	1:3000
Mouse monoclonal anti-N-cadherin antibody	Santa Cruz Biotechnology, TX, sc-393933	1:200	1:1000
Rabbit polyclonal anti-α-SMA antibody	Abcam, Cambridge, UK, ab5694	1:500	-
Mouse monoclonal anti-TNF alpha antibody	Abcam, Cambridge, UK, ab1793	1:500	-
Rabbit polyclonal anti-NF-kB p65 (Ser536) antibody	Bioss, MA, BS-092R	1:500	1:3000
Mouse monoclonal anti-p-NF-kB p65 antibody	Santa Cruz Biotechnology, TX, sc-8008	1:100	1:1000
Rabbit polyclonal anti-survivin antibody	R & D systems, MA, AF886	-	1:3000
Rabbit polyclonal anti- anti-XIAP antibody	Abcam, Cambridge, UK, ab137392	-	1:3000
Mouse monoclonal anti-HIF-1α antibody	Santa Cruz Biotechnology, TX, sc-53546	1:100	1:2000
Goat anti-rabbit HRP	Promega, WI, W4018	1:500	1:10,000
Donkey anti-mouse HRP	Abcam, Cambridge, UK, ab98799	1:500	1:10,000
Goat Anti-human IgG (H+L)-FITC, affinity purified	Jackson Immune-Research Laboratories Inc., PA, USA; 109-095-088	1:500	-
Mouse monoclonal anti-actin antibody	Millipore, Billerica, MA, MAB1501	-	1:40,000
Donkey anti-mouse-Alexa fluor 488	Abcam, Cambridge, UK, ab150109	1:500	-

Goat anti-rabbit IgG-Alexa fluor 555	Abcam, Cambridge, UK, ab150086	1:750	-
Goat anti-rabbit Alexa fluor 488	Abcam, Cambridge, UK, ab150078	1:750	-
Goat anti-mouse Alexa fluor 555	Abcam, Cambridge, UK, ab150114	1:750	-

Table S2. Gene specific q-PCR primers used in the study

No	Gene name	Primers
1.	*CSTB*	Forward GTCCCAGCTTGAAGAGAAAGAA<br>Reverse GACGTGCACCTTGATGAAGTA
2.	*ANXA3*	Forward CCAGACCTGGTCATCAATGTAG<br>Reverse GCATAACTCTCTCCTTCTCTTGG
3.	*CA8*	Forward CTGTGACTGAAATCCTCCAAGA<br>Reverse CAGCAGAGGGTCTGGTAATAAA
4.	*SCAMP4*	Forward CATCTTCGGAGCCCAGTTT<br>Reverse GGCTGTACTGGAAGAATCCAA
5.	*SYTL2*	Forward CTACCTCTCTCTGGGCTTCTAT<br>Reverse ATCTTCAGGAGACGCTCGTA
6.	*CD38*	Forward TGAGACATGTAGACTGCCAAAG<br>Reverse CCCAACTTCATTAGTGGCTGATA
7.	*SUSD2*	Forward GGACTTCTGCCTGGAGATATTG<br>Reverse GGTCTGGATGCTGTCCTTAAA
8.	*TNS4*	Forward TCTCACTGGAGAGCCTCAAT<br>Reverse GGCTTCAGATTCCTCCTTCTTT
9.	*ADGRG*	Forward CGACATGCTGGGAGATTACA<br>Reverse GGTGACAGAAGTGGCTAACA
10.	*ANXA8L1*	Forward CAAGTCCTTCAAGGCTCAGTT<br>Reverse CCTTGGCTTCGTATCTGTATGG
11.	*Ki-67*	Forward GAAAGAGTGGCAACCTGCCTTC<br>Reverse GCACCAAGTTTTACTACATCTGCC
12.	*Caspase3*	Forward GGAAGCGAATCAATGGACTCTGG<br>Reverse GCATCGACATCTGTACCAGACC
13.	*p53*	Forward CCTCAGCATCTTATCCGAGTGG<br>Reverse TGGATGGTGGTACAGTCAGAGC

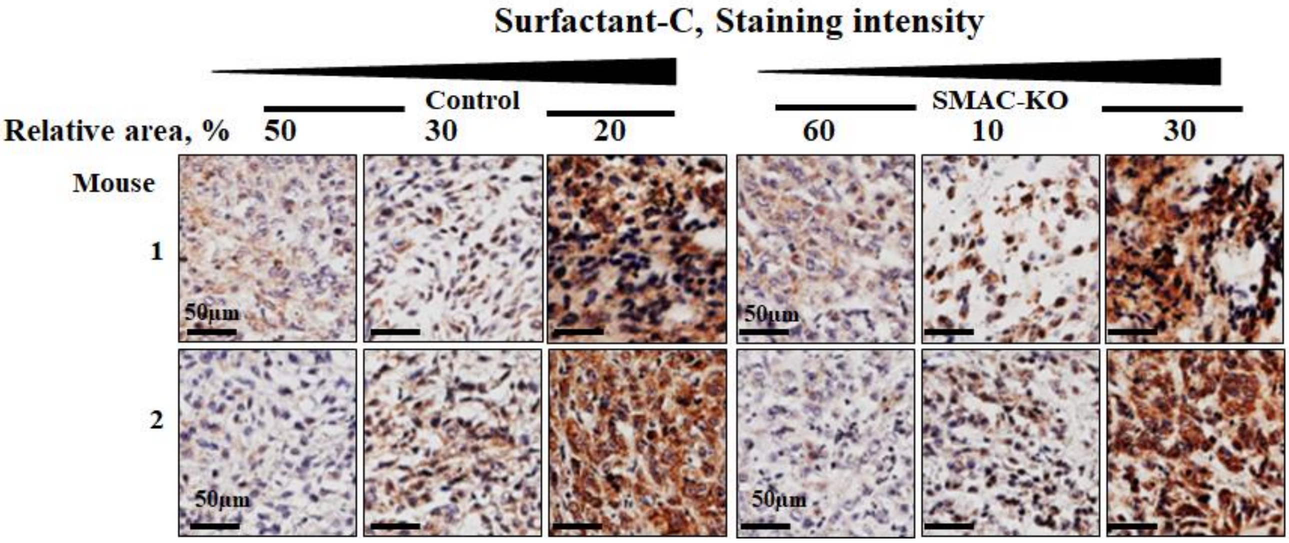

Fig. S1. Surfactant-C staining intensity in control and SMAC-OK tumors
Representative IHC staining of Surfactant-C in tumor sections from Control (n = 3 mice) and SMAC-KO (n = 3) mice. Percentage represents the relative area stained for Surfactant-C at the indicated intensity presented in the scale at the top of the figure.

Table S3. Alterations in the expression of lipids and lipid-signaling molecule-related human protein SMAC-KO A549 in human cancer cells
LC-HR MS/MS analysis was performed as described in the Materials and Methods section. Differentially expressed proteins (*p*-value <0.05, FC |1.5|) were filtered, and those proteins differentially expressing SMAC-KO cells are presented. For each protein, the name, fold change (FC), and *p*-value, as well as its function, subcellular localization and relevance to cancer are indicated.

No.	Protein name (UniProtKB)	Proposed function (Cellular localization)	FC (*p*-value)	Relation to cancer
1.	**Ethanolamine kinase 1** (ETNK1) (*Q9HBU6*)	Ethanolamine phosphorylation. (cytoplasm)	+1.79 (0.0275)	Potential diagnostic marker and therapeutic target in breast and pancreatic cancers [2]
2.	**Myotubularin-related protein 1** (MTMR1) (*Q13613*)	A tyrosine/dual-specificity phosphatase, with phosphatidy-linositol 3-monophosphate (PI$_3$P) and/or phosphatidylinositol 3,5-bisphosphate (PI$_{3,5}$P$_2$) as substrates (plasma membrane, cytoplasm)	-1.54 (0.0425)	Not reported
3.	**3-ketodihydro-sphingosine reductase** (KDSR),(*Q06136*)	Catalyzes the reduction of 3-ketodihydrosphingosine to dihydrosphingosine (ER)	-1.56 (0.0254)	Not reported
4.	**Type 1 phosphatidyl inositol 4,5-bisphosphate 4-phosphatase** (PIP4P1),(*Q86T03*)	Catalyzes the hydrolysis of phosphatidyl-inositol-4,5-bisphosphat to phosphatidyl inositol-4-phosphate (endosome, lysosome, plasma membrane)	-1.56 (0.0253)	Not reported
5.	**Methylmalonyl-CoA mutase, mitochondrial** (MMUT) (P22033)	Involved in the degradation of several amino acids, odd-chain fatty acids, and cholesterol via propionyl-CoA to the tricarboxylic acid cycle (mitochondria)	-1.59 (0.0284)	Not reported
6.	**Phospholipid phosphatase 2** (PLPP2) (O43688)	Phospholipid phosphatase, dephosphorylates a variety of glycerolipid and sphingolipid phosphate esters (ER, endosome, plasma membrane)	-1.63 (0.0251)	Not reported

7.	**Phospholipid phosphatase 3** (PLPP3) (O14495)	A cell-surface glycoprotein converting phosphatidic acid to diacylglycerol, hydrolyzing extracellular lysophosphatidic acid (ER, golgi, plasma membrane)	-1.69 (0.0129)	Promotes proliferation and tumorigenesis, activates Ca^{2+}- channel in lung carcinoma cells [3]
8.	**Sn1-specific diacyl-glycerol lipase beta** (DAGLB),(Q8NCG7)	Catalyzes the hydrolysis of diacylglycerol to 2-arachidonoyl-glycerol (cell membrane)	-1.71 (0.0319)	Not reported
9.	**Lysophospholipid acyltransferase** (LPCAT4) (*Q643R3*)	Converts lysophosphatidyl ethanolamine to phosphatidyl ethanolamine, 1-alkenyl-lysophatidyl-ethanolamine to 1-alkenyl-phosphatidyl ethanolamine, lysophosphatidyl glycerol and alkyl-lysophosphatidyl choline to phosphatidyl-glycerol and alkyl-phosphatidylcholine, respectively (ER)	-1.73 (0.0306)	Overexpressed and responsible for PC accumulation in CRC [4]
10.	**Very-long-chain (3R)-3-hydroxyacyl-CoA dehydratase 2** (HACD2) (*Q6Y1H2*)	Long-chain fatty acid metabolism. Adding two carbons to the chain of long- and very long-chain fatty acids. Catalyzes the dehydration of the 3-hydroxyacyl-CoA intermediate into trans-2,3-enoyl-CoA, within each cycle of fatty acid elongation (ER)	-1.89 (0.023)	Not reported
11.	**Epoxide hydrolase 1** (EPHX1) (P07099)	Biotransformation enzyme—plays a role in the metabolism of endogenous lipids such as epoxide-containing fatty acids (ER)	-2.04 (0.0292)	Overexpressed in breast and liver cancer and correlated with poor outcome in patients receiving tamoxifen [5]
12.	**Acetyl-coenzyme A synthetase 2-like, mitochondrial** (ACSS1) (*Q9NUB1*)	Catalyzes the synthesis of acetyl-CoA from short-chain fatty acids (mitochondria)	-2.6 (0.0123)	Promotes acetate utilization and cancer cell growth under metabolic stress [6]
13.	**Phospholipid hydro-peroxide glutathione peroxidase** (GPX4) (P36969)	Essential antioxidant peroxidase that reduces phospholipid hydroperoxide (mitochondria, cytoplasm)	-2.74 (0.00399)	Plays a role in protecting cancer cells from cytotoxicity [7]
14.	**Monoglyceride lipase** (MGLL) (*Q99685*)	Converts mono-acylglycerides to free fatty acids and glycerol-l (cytosol)	-3.05 (0.00516)	Highly expressed in aggressive human cancer cells and primary tumors. Regulates a fatty acid network, enriched in oncogenic signaling lipids that promotes migration, invasion, survival, and in-vivo tumor growth [8]
15.	**Glycosylphosphatidyl inositol anchor attachment 1 protein** (GPAA1), (O43292)	Involved in the pathway glycosylphosphatidylinositol-anchor biosynthesis, involved in glycolipid biosynthesis (ER)	-4.14 (0.0165)	Overexpressed and induces tumorigenesis in several types of cancer [9]
16.	**Diablo homolog, mitochondrial** (DIABLO)(Q9NR28) **SMAC**	Inhibitor of inhibitors of apoptosis (IAP) to activate caspase, and thereby, apoptosis, and we showed it to regulate phospholipid synthesis (mitochondria, nucleus)	-13.4 (0.0276)	Overexpressed in several types of cancer and potential drug target [10]
17.	**Annexin A8-like protein 1** (ANXA8L1) (Q5VT79)	Calcium-dependent phospholipid binding, may function as an anticoagulant that indirectly inhibits the thromboplastin-specific complex (cytoplasm)	-16.5 (0.000254)	Upregulated in pancreatic cancer [11]

Table S4. Alterations in the expression of transport and trafficking-related human protein SMAC-KO A549 human cancer cells

LC-HR MS/MS analysis was performed as described in the Materials and Methods section. Differentially expressed proteins (*p*-value <0.05, FC |1.5|) were filtered, and those proteins differentially expressing SMAC-KO cells are presented. For each protein, the name, fold change (FC), and *p*-value, as well as its function, subcellular localization, and relevance to cancer are indicated.

No.	Protein name (UniProtKB)	Proposed function (Cellular localization)	FC (*p*-value)	Relation to cancer
	**Transport**			
1.	**Solute carrier family 12 member 2 (**SLC12A2) (P55011)	Mediates sodium and chloride reabsorption. Plays a vital role in the regulation of ionic balance and cell volume (plasma membrane)	+2.38 (0.0137)	Overexpression in colon cancer [12]
2.	**Tetraspanin-13** (TSPAN13) **(**O95857)	Cell surface protein, regulates Ca^{2+} channel (CaV2.2) activity in synaptic membrane (plasma membrane)	+1.87 (0.00993)	Downregulation in prostate cancer [13]
3.	**Large subunit GTPase 1 homolog (**LSG1) (Q9H089)	Nuclear export of the 60S ribosomal subunit (cytoplasm, ER, nucleus)	+1.77 (0.0321)	Not reported
4.	**ADP/ATP translocase 3 (SLC25A3) (P12236)**	antiporter of ATP/ADP at the IMM (mitochondria)	+1.59 (0.0375)	Required for some apoptoti cell death in breast cancer [14]
5.	**Translocon-associated protein subunit gamma (**SSR3) (Q9UNL2)	Subunit of the translocon-associated protein (TRAP) complex which mediates protein translocation across the ER (ER)	-1.78 (0.0257)	Overexpressed and prognostic biomarker of metastatic in CRC [15]
6.	**V-type proton ATPase subunit α isoform 3 (**V-ATPase**)** (Q13488)	Subunit of the V-ATPases, a multimeric enzyme that catalyzes the translocation of protons across membranes (endosomes, lysosomes)	-1.92 (0.00782)	High expression level of ATP6V0A1 correlated with improved survival in CRC patients [16]
7.	**SEC14-like protein 2** (SEC14L2) (O76054)	Transports hydrophobic molecules at different cellular sites (nucleus, cytoplasm)	-1.93 (0.0279)	Reduced expression in human breast cancer [17]
8.	**Solute carrier family 12 member 9** (SLC12A9) **(**Q9BXP2)	Cation—chloride, specifically potassium—chloride symporter (plasma membrane)	-1.98 (0.0315)	Not reported
9.	**Non-imprinted in Prader-Willi/ Angelman syndrome region protein 1 (**NIPA1**)** (Q7RTP0)	Mg^{2+} transporter and other divalent cations such as Fe^{2+}, Sr^{2+}, Ba^{2+}, Mn^{2+}, and Co^{2+} (endosome, plasma membrane)	-1.99 (0.0356)	Not reported
10.	**Solute carrier family 2, facilitated glucose transporter member 3** (SLC2A3) (P11169)	Glucose transporter, but also transports 2-deoxyglucose, galactose, mannose, xylose, and fucose across the cell membrane (cell membrane)	-2.94 (0.0133)	Upregulation is associated with decreased survival in colorectal cancer patients [18]
11.	**Apolipoprotein L2** (APOL2) (Q9BQE5)	May function in the movement of lipids and binding of lipids to organelles (cytoplasm)	-2.99 (0.00109)	Overexpressed in cervical cancer [19]
12.	**Solute carrier family 35 member B1** (SLC35B1) (P78383)	Antiporter, transporting nucleotide sugars and adenosine 3'-phospho 5'-phosphosulfate into the golgi from cytoplasm and vice versa (ER)	-3.48 (0.0122)	Not reported
13.	**Niemann-Pick disease, type C1 (**NPC1) (O15118)	A trans-membrane protein facilitating cholesterol transpt into	-4.74 (0.00655)	NPC1 depletion in several cancer cell lines inhibits cel

		endosomes and lysosomes (lysosome, endosome)		proliferation and migration [20]
14.	**Vacuolar protein sorting-associated protein 52 homolog** (VPS52) **(**Q8N1B4)	Acts as a component of the golgi-associated retrograde protein complex, involved in retrograde transport from early and late endosomes to the trans-golgi network (golgi, endosome)	-5.16 (0.000139)	In gastric cancer, induces apoptosis via cathepsin D [21]
	**Trafficking**			
15.	**Ras-related protein Rab-8A (**RAB8A) (P61006)	Small GTPases, key regulators of intracellular membrane trafficking, vesicle fusion with membranes (golgi, plasma membrane, cytoplasm, cytoskeleton)	-1.52 (0.0364)	Overexpressed and a potential biomarker for endometrial cancer [22]
16.	**Yip1 Interacting Factor Homolog A** (YIF1A) (O95070)	Possible role in transport of vesicles between ER and golgi (ER, golgi)	-1.73 (0.0367)	Not reported
17.	**CLN3 lysosomal/endosomal transmembrane protein** (CLN3) (Q13286)	A lysosomal/endosomal membrane protein; its precise function is not known. May be involved in endocytic trafficking (endosome, plasma membrane, golgi)	-1.75 (0.0226	Not reported
18.	**ER-golgi intermediate compartment protein 1 (**ERGIC1) (Q969X5)	A cycling membrane protein of the ER-golgi intermediate compartment (ERGIC), interacting with other members of this protein family to increase their turnover (ER, golgi)	-2.03 (0.00946)	A potential drug target in prostate cancer [23]
19.	**Syndecan binding protein** (SDCBP) (O00560)	It is a PDZ-domain-containing protein, interacts with many partners, and regulates transmembrane-receptor trafficking, (nucleus, ER, cytoplasm, plasma membrane)	-2.12 (0.0394)	Induces immune evasion in triple-negative breast cancer [24] and head and neck squamous cell carcinoma [25]
20.	**Translocating chain-associated membrane protein 1** (TRAM1) (Q15629)	Influences protein glycosylation and facilitates the translocation of secretory proteins across the ER (ER)	-3.06 (0.0178)	Not reported
21.	**Synaptotagmin-like protein 2** (SYTL2) (Q9HCH5)	Plays a role in cytotoxic granule exocytosis in lymphocytes and required for cytotoxic granule docking at the immunologic synapse (plasma membrane, cytoplasm)	-5.91 (0.039)	Overexpressed associated with invasion and metastasis [26]
22.	**Secretory carrier-associated membrane protein 4** (SCAMP4) (Q969E2)	Widely distributed, integral membrane proteins implicated in membrane trafficking (plasma membrane)	-8.12 (0.00049)	Not reported

Table S5. Alterations in the expression of metabolism-related human proteins SMAC-KO A549 human cancer cells

LC-HR MS/MS analysis was performed as described in the Materials and Methods section. Differentially expressed proteins (*p*-value <0.05, FC |1.5|) were filtered and those proteins differentially expressed SMAC-KO cells are presented. For each protein, the name, fold change (FC), and *p*-value, as well as its function, subcellular localization and relevance to cancer are indicated.

No.	Protein name (UniProtKB)	Proposed function (Cellular localization)	FC (*p*-value)	Relation to cancer
1.	**Carbonic anhydrase-related protein** (CA8) (P35219)	CA8 affecting IP_3 binds to its receptor IP_3R1 in the ER, thereby modulating Ca^{2+} signaling [27]. Has a role in brain development, mainly expressed in the Purkinje cells of the cerebellum and in a wide variety of other tissues (cytoplasm)	+6.77 (0.0000965)	Ectopic overexpression of CA8 reduces the growth of lung cancer cells [28]. Reduced expression is associated with progression of renal cell carcinoma [29]. However, it is overexpressed in some cancer types [30]
2.	**Liver carboxylesterase 1** (CES1) (P23141)	Involved in the detoxification of xenobiotics and in the activation of ester and amide pro-drugs (ER)	+5.42 (0.00037)	Expressed and mutated specifically in cancer cells [31]
3.	**Amine oxidase** [flavin-containing] **(**AMAO8) (P21397)	Catalyzes the oxidative deamination of biogenic and xenobiotic amines (OMM, cytosol)	+4.59 (0.00674)	Overexpressed in breast cancer [32]
4.	**Dolichyl pyro-phosphate Man9-GlcNAc2 alpha-1,3 glucosyltransferase** (ALG6) (Q9Y672)	Adds the first glucose residue to the lipid-linked oligosaccharide precursor for N-linked glycosylation (ER)	+2.95 (0.00124)	Not reported
5.	**Glycine dehydrogenase (decarboxylating), mitochondrial (**GLDC) (P23378)	The glycine cleavage system catalyzes the degradation of glycine (mitochondria)	+1.94 (0.0252)	Associated with better survival, and is a factor for the favorable prognosis of hepatocellular carcinoma patients [33]
6.	**Trans-3-hydroxy-L-proline dehydratase** (L3HYPDH) (Q96EM0)	In proline metabolism catalyzes the dehydration of trans-3-hydroxy-L-proline to Delta1-pyrroline-2-carboxylate (cytoplasm)	+1.85 (0.0226)	Not reported
7.	**Peptidyl-prolyl cis-trans isomerase (**FKBP4) Cyclophilin (Q02790)	Immunophilin protein with PPIase and co-chaperone activities, acts also as a regulator of microtubule dynamics (mitochondria, nucleus, cytoplasm, cell membrane)	+1.78 (0.0161)	Overexpressed in castration-resistant prostate cancer and is a potential therapeutic target [34]
8.	**Phosphoribosylformylglycinamidine synthase (**PFAS) (O15067	Involved in purine biosynthetic pathway, and catalyzes the ATP-dependent conversion of formyl-glycinamide ribonucleotide and glutamine to yield formyl-glycine-amidine ribonucleotide (cytoplasm)	+1.78 (0.0109)	Increased activity in cancer cells [35]
9.	**Bifunctional methylene tetrahydr of olate-dehydrogenase /cyclohydrolase** (MTHFD2) (P13995)	NAD-specific bifunctional enzyme with methylene-tetrahydrofolate dehydro-genase and methenyl-tetrahydrofolate cyclohydrolase activities (mitochondria)	+1.77 (0.0386)	Critical for cancer cell stemness and resistant to drugs including gefitinib [36]

10.	**tRNA pseudo-uridine synthase A** (PUS1) (Q9Y606)	Uridine metabolism involved in regulating nuclear receptor activity through pseudo-uridylation of SRA1 RNA (nucleus, mitochondria)	+1.73 (0.0166)	Overexpressed in hepatocellular carcinomas, lung, and prostate cancers [37]
11.	**DnaJ homolog sub-family B member 4** DNAJB4 (Q9UDY4)	Stimulates ATP hydrolysis and the folding of unfolded proteins mediated by HSPA1A/B (cytoplasm, cell membrane)	+1.66 (0.0386)	Not reported
12.	**ATP-dependent 6-phosphofructokinas, muscle type** (PFKM) (P08237)	Catalyzes the phosphorylation of D-fructose 6-phosphate to fructose 1,6-bisphosphate by ATP (cytoplasm)	+1.55 (0.0279)	Not reported
13.	**Glutamate-cysteine ligase catalytic subunit** (GCLC) (P48506)	The first rate-limiting enzyme of glutathione synthesis (cytosol, mitochondria)	+1.54 (0.0413)	Overexpressed in lung cancer and associated with cisplatin resistance [38]
14.	**Glutathione hydrolase 1 proenzyme** (GGT1) (P19440)	Glutathione metabolism that cleaves th γ-glutamyl bond of extracellular glutathione (gamma-Glu-Cys-Gly), glutathione conjugates, and other γ-glutamyl compounds (ceil membrane)	-1.52 (0.0316)	Not reported
15.	**N(G),N(G)-dimethylarginine dimethylamino-hydrolase 1** (DDAH1) (O94760)	Hydrolyzes N(G), N(G)-dimethyl-L-arginine, and N(G)-mono-methyl-L-arginine which act as inhibitors of NOS, therefore, regulating nitric oxide generation (cytosol)	-1.54 (0.0335)	Enhances tumor growth and angiogenesis [39]
16.	**Serine hydroxyl methyltransferase** (SHMT1) (P34896)	Interconversion of serine and glycine (cytoplasm)	-1.58 (0.0196)	Not reported
17.	**Aldo-keto reductase family 1 member D1 (**AKR1D1) (P51857)	Catalyzes the stereospecific NADPH-dependent reduction of the C4-C5 double bond of bile acid intermediates and steroid hormones (cytoplasm)	-1.6 (0.0338)	Not reported
18.	**Phosphoglycerate kinase 1** (PGK1) (P00558)	Glycolytic enzyme catalyzes the reversible transfer of a phosphate group from 1,3-bisphospho-glycerate (1,3-BPG) to ADP, producing 3-phospho-glycerate (3-PG) and ATP (cytoplasm)	-1.63 (0.0439)	Overexpressed in liver cancers and promotes oncogenesis and chemoresistance [40]
19.	**Polypeptide N-acetylgalactosaminyltransferase 1** (GALNT1) (Q10472)	Catalyzes the initial reaction in O-linked oligosaccharide biosynthesis, the transfer of an N-acetyl-D-galactosamine residue to a serine or threonine residue on the protein receptor (Golgi)	-1.69 (0.0433)	Aberrant glycosylation due to mutations in the enzyme was found in melanoma, ovarian, and bladder cancers [41]
20.	**Aldehyde dehydrogenase family 3 member B1** (ALDH3B1) (P43353)	Oxidizes medium and long chain saturated and unsaturated aldehydes (cell membrane)	-1.78 (0.0105)	Overexpressed and a marker for CRC [42]
21.	**NADPH-cytochrome P450 reductase** (POR) (P16435)	Responsible for transferring electrons to many naturally occurring electron acceptors, including cytochrome P450 enzymes and cytochrome-b5 (ER)	-1.81 (0.0166)	Enhances doxorubicin toxicity in breast cancer [43]

22.	**All-trans-retinol 13,14-reductase (**RETSAT) (Q6NUM9)	Catalyzes the saturation of all-trans-retinol to all-trans-13,14-dihydroretinol (ER)	-1.86 (0.0135)	Not reported
23.	**ADP-ribose glycohydrolase ARH3 (**ADPRS) (Q9NX46)	ADP-ribose glycohydrolase hydrolyzes the scissile alpha-O-linkage attached to the anomeric C1" position of ADP-ribose and acts on different substrates (mitochondria, nucleus)	-1.95 (0.0148)	Overexpressed in breast cancer [44]
24.	**UDP-N-acetyl hexosamine pyro phosphorylase-like protein 1** (UAP1L1) (Q3KQV9)	UDP-N-acetylglucosamine biosynthetic process (cytoplasm)	-2.68 (0.0174)	A critical factor for protein O-GLCN-acylation and cell proliferation in human hepatoma cells [45]
25.	**Protein mono-ADP-ribosyltransferase (**PARP14) (Q460N5)	Mono-ADP-ribosylation of glutamate residues on target proteins (nucleus, cytoplasm)	-3.0 (0.00689)	A novel drug target for diffuse large B-cell lymphoma, multiple myeloma, prostate cancer, and hepatocellular carcinoma [46]
26.	**Sulfotransferase 1A4 (**SULT1A4) (P0DMN0)	Catalyzes the sulfate conjugation of phenolic monoamines (cytoplasm)	-4.03 (0.0786)	Not reported
27.	**Argininosuccinate lyase (**ASL) (P04424)	Involved in step 3 that synthesizes L-arginine from L-ornithine and carbamoyl phosphate (cytoplasm)	-4.3 (0.00984)	Potential therapeutic target in breast cancer and CRC [47, 48]
28.	**UDP-glucuronosyl transferase 1-7** (UGT1A7) (Q9HAW7)	Metabolism of xenobiotics and endogenous compounds (ER)	-13.0 (0.000146)	Overexpressed, associated with biochemical recurrence in prostate cancer [49]

Table S6. Alterations in the expression of ECM and structural proteins in SMAC-KO A549 human cancer cells

LC-HR MS/MS analysis was performed as described in the Materials and Methods section. Differentially expressed proteins (*p*-value <0.05, FC |1.5|) were filtered, and those proteins differentially expressing SMAC-KO cells are presented. For each protein, the name, fold change (FC), and *p*-value, as well as its function, subcellular localization, and relevance to cancer are indicated.

**No.**	**Protein name (UniProtKB)**	**Proposed function (Cellular localization)**	**FC (*p*-value)**	**Relation to cancer**
1.	**Fibronectin (**FN1) (P02751)	Involved in cell adhesion and motility, wound healing, maintenance of cell shape, and osteoblast compaction (ER)	+5.18 (0.0381)	Overexpressed in the stroma of squamous cell carcinoma tumors [50, 51]
2.	**Collagen alpha-2(V) chain** (COL5A2) *(P05997*)	A connective tissue component that binds to DNA, heparin sulfate, heparin, insulin, and thrombospondin. A key protein in the assembly of tissue-specific matrices (ER)	+4.98 (0.000195)	Overexpressed in bladder cancer [52]
3.	**Unconventional myosin-Ixb** (MYO9B) (*Q13459*)	Binds actin with high affinity in both the absence and presence of ATP (cytoskeleton)	+2.56 (0.0239)	Overexpressed in lung cancer [53]
4.	**Ensconsin** (MAP7) (*Q14244*)	Microtubule-stabilizing protein functions in reorganization of microtubules during polarization and differentiation of epithelial cells (plasma membrane)	+2.31 (0.00253)	Overexpressed in colon cancer [54]

5.	**Gamma-adducin** (ADD3) (*Q9UEY8*)	Membrane-cytoskeleton-associated protein that promotes the assembly of the spectrin-actin network and in actin filament capping (cell membrane)	+1.85 (0.0352)	Highly phosphorylated and redistributed during renal tumor progression [55]
6.	**Tubulin beta-4A chain** (TUBB4A) (*P04350*)	A major constituent of microtubules, binds 2 GTP, one in the beta chain and the second in the alpha chain (cytoskeleton)	+1.77 (0.0163)	Not reported
7.	**CLIP-associating protein 1** (CLASP1) (Q7Z460)	Microtubule assembly protein that promotes the stabilization of dynamic microtubules and is involved in the nucleation of non-centrosomal microtubules originating from the trans-Golgi network (cytoskeleton, nucleus)	+1.68 (0.0132)	Not reported
8.	**Integrin alpha-2** (ITGA2) (P17301)	Cell receptor for laminin, collagen, collagen C-propeptides, fibronectin, and E-cadherin. Responsible for adhesion of platelets and other cells to collagens (cell membrane, cytoplasm)	+1.68 (0.0256)	Plays a direct role in cancer progression, tumor cell survival, angiogenesis, and metastasis [13]
9.	**SCY1-like protein 2** (SCYL2) (*Q6P3W7*)	Component of adaptor protein complex 2 containing clathrin-coated structures at the plasma membrane or endocytic-coated vesicles (cell membrane)	+1.61 (0.0161)	Not reported
10.	**Pleckstrin homology-like domain family B member 2** (PHLDB2) (Q86SQ0)	Seems to be involved in the assembly of the postsynaptic apparatus. (cytoplasm, cell membrane)	-1.57 (0.0415)	Higher expression is associated with shorter overall survival and metastasis-free colon cancer patients. Inhibition causes reduced cell invasion and migration. [56]
11.	**Microtubule cross-linking factor 1** (MTCL1) (Q9Y4B5)	Microtubule-associated factor involved in the late phase of epithelial polarization and microtubule dynamics regulation. Functions in maintenance of non-centrosomal microtubule bundles at the lateral membrane in polarized epithelial cells (cytoskeleton, plasma membrane)	-1.62 (0.0165)	Overexpressed, associated with cancer development, as breast, colon, and lung cancer, and glioblastoma [57]
12.	**Calmin** (CLMN) (Q96JQ2)	May bind actin and connect the nucleus to the actin cytoskeleton (cell membrane)	-1.79 (0.011)	Upregulated in human, breast cancer, myeloid leukemia cells, and neuroblastoma [58]
13.	**PDZ domain-containing protein 8** (PDZD8) (*Q8NEN9*)	Molecular tethering protein that connects the ER and mitochondria membranes (ER membrane)	-1.85 (0.0089)	Not reported
14.	**Reticulon-4** (RTN4) (Q9NQC3)	Required for the formation and stabilization of ER tubules and regulate membrane morphogenesis in the ER by promoting tubular ER production (ER)	-1.88 (0.0482)	Involved in prostate cancer progression and is a therapeutic target [59]
15.	**Transforming growth factor-beta-induced protein ig-h3** (TGFBI) (Q15582)	Plays a role in cell adhesion and in epithelial-to-mesenchymal transition (extra cellular matrix)	-2.94 (0.0343)	High expression in lung cancer and associated wit[illegible] poor prognosis and therapeutic resistance to immune checkpoint inhibitors [60]
16.	**Smoothelin** (SMTN) (*P53814*)	Smooth muscle-specific contractile protein expressed only by fully differentiated, and not by proliferative or	-3.01 (0.0117)	A biomarker for bladder urothelial carcinoma [61]

		noncontractile, smooth muscle cells and myofibroblasts (cytoskeleton)		
17.	**Claudin-2** (CLDN2) (P57739)	Plays a major role in tight junction-specific obliteration of the intercellular space through calcium-independent cell-adhesion activity (cell membrane)	-3.85 (0.0478)	Promotes progression of several type of cancers including lung, breast, and CRC [62-64]
18.	**Plakophilin-2** (PKP2) (Q99959)	Cell adhesion that is most widespread desmosome plaque protein of epithelial and non-epithelial tissues such as myocardium and lymph node follicles and of cultured cells (nucleus, desmosomes)	-4.26 (0.00957)	A novel activator of the EGFR signaling pathway and a potential new drug target for inhibiting tumor growth [65]
19.	**Protein-glutamine gamma-glutamyl-transferase 2** (TGM2) (*P21980*)	Catalyzes the cross-linking of proteins, such as WD repeat-containing protein, important for extracellular matrix integrity (ER, cytosol, cell membrane, mitochondria)	-5.83 (0.000483)	A potential molecular marker for chemotherapeutic drug sensitivity in cancer [66]
20.	**Adhesion G-protein coupled receptor G1** (ADGRG1) (*Q9Y653*)	Receptor involved in cell adhesion and probably in cell–cell interactions (plasma membrane)	-14.4 (7.78E-5)	Overexpressed in various cancer types functions in adhesion, migration, and metastasis [67]
21.	**Tensin-4** (TNS4) (*Q8IZW8*)	May be involved in cell migration, cartilage development, and in linking signal transduction pathways to the cytoskeleton (cytoskeleton)	-16.7 (0.00596)	Upregulated in colorectal, lung, ovarian, and gastric cancers, and correlates with tumor progression [68]

Table S7. Alterations in the expression of signaling pathways and development- and differentiation-related proteins in SMAC-KO A549 human cancer cells

LC-HR MS/MS analysis was performed as described in the Materials and Methods section. Differentially expressed proteins (*p*-value <0.05, FC |1.5|) were filtered, and those proteins differentially expressing SMAC-KO cells are presented. For each protein, the name, fold change (FC), and *p*-value, as well as its function, subcellular localization, and relevance to cancer are indicated.

No.	Protein name (UniProtKB)	Proposed function (Cellular localization)	FC (*p*-value)	Relation to cancer
1.	**Protein strawberry notch homolog 1** (SBNO1) (A3KN83)	A novel chromatin factor of the helicase superfamily 2 (nucleus)	+3.44 (0.0157)	Not reported
2.	**High mobility group protein** (HMGI-C) (P52926)	Transcriptional regulator, functions in cell cycle regulation (nucleus)	+3.34 (0.00075)	Overexpressed in breast cancer [69]
3.	**TM2 domain-containing protein 3** (TM2D3) (Q9BRN9)	May have regulatory roles in cell death or proliferation signal cascades (cell membrane)	+3.02 (0.00213)	Not reported
4.	**Adhesion G-protein coupled receptor G6** (ADGRG6) (Q86SQ4)	Essential for normal differentiation of promyelinating Schwann cells and for normal myelination of axons (cell membrane)	+3.01 (0.0225)	Not reported
5.	**Serine/threonine-protein kinase** (VRK2) (Q86Y07)	A kinase enzyme that phosphorylates serine or threonine. Regulates several signal transduction pathways (ER, mitochondria)	+2.29 (0.00266)	VRK2 inhibits mitogen-activated protein kinase signaling and inversely correlates with ErbB2 in human breast cancer [70]

6.	**YY1-associated factor** 2YAF2 (Q8IY57)	Binds to MYC and inhibits MYC-mediated transactivation (nucleus)	+2.02 (0.0117)	Interacts with Myc N in neuroblastoma [71]
7.	**Myocyte-specific enhancer factor 2D** (MEF2D) (Q14814)	Transcriptional activator, functions in skeletal and cardiac muscle development, and in neuronal differentiation and survival (nucleus)	+2.00 (0.0378)	Overexpressed in aggressive leiomyosarcoma [72]
8.	**Nuclear factor NF-kappa-B p100 subunit** (NFKB2) (Q00653)	Pleiotropic transcription factor, present in almost all cell types (nucleus, cytoplasm)	+1.93 (0.00895)	Contributes to development of malignant tumors [73]
9.	**Zinc finger protein 384** (ZNF384) (Q8TF68)	Transcription factor that binds the consensus DNA sequence [GC]AAAAA. Seems to regulate the promoters of MMP1, MMP3, MMP7, and COL1A1 (nucleus)	+1.8 (0.0124)	A poor prognostic predictor that promotes cell growth by upregulating the expression of Cyclin D1 in hepatocellular carcinoma [74]
10.	**Integrin alpha-5** (ITGA5) (P08648)	Fibronectin and fibrinogen receptor. Upstream receptor for FAK, RRC, SFK signaling (cell membrane)	+1.75 (0.027)	Implicated in cell proliferation, differentiation, and migration of cancer cells [75]
11.	**Serine/threonine-protein kinase** (WNK1) (Q9H4A3)	Regulation of survival, electrolyte homeostasis, cell signaling, and proliferation (cytoplasm)	+1.75 (0.0106)	Potential therapeutic targets for GBM stem-like cells [76]
12.	**ETS domain-containing transcription factor** (ERF)(P50548)	Potent transcriptional repressor that binds to the H1 element of the Ets2 promoter (nucleus)	+1.65 (0.0196)	Plays a major role in prostate oncogenesis and is a potential drug target [17]
13.	**Zinc finger protein 512** (ZNF512) (Q96ME7)	Involved in a variety of processes, including regulation of transcription (nucleus)	+1.62 (0.0301)	Not reported
14.	**Melanoma-associated antigen D1** (MAGED1) (Q9Y5V3)	Inhibits cell cycle progression, and facilitates NGFR-mediated apoptosis (nucleus, cell membrane, cytoplasm)	+1.59 (0.0329)	Overexpression suppresses cell migration, invasion, and adhesion. Disrupts actin cytoskeleton rearrangement induced by hypoxia and down-regulated HIF-1expression [77]
15.	**Leucine-rich repeat-containing protein 34** (LRRC34) (Q8IZ02)	Regulation of pluripotency in embryonic stem cells. Promotes stem cell proliferation *in vitro* (nucleus, cytoplasm)	-1.61 (0.0331)	Not reported
16.	**SLIT-ROBO Rho GTPase-activating protein 1** (SRGAP1) (Q7Z6B7)	GTPase-activating protein for RhoA and Cdc42 small GTPases (cytoplasm)	-1.65 (0.0187)	Overexpressed in gastric carcinogenesis and plays an oncogenic role via activating the Wnt/β-catenin pathway [78]
17.	**Transcription factor COE2** (EBF2) (Q9HAK2)	Transcription factor that activates the decoy receptor for RANKL, TNFRSF11B, which in turn regulates osteoclast differentiation (nucleus)	-1.69 (0.0275)	Not reported
18.	**Rab-like protein 3** (RABL3) (Q5HYI8)	Nucleotide binding protein involved in Rab protein signal transduction. (cell membrane)	-1.75 (0.0137)	May be involved in cancer progression [79]
19.	**Ras-associated and pleckstrin homology domains-containing protein 1** (RAPH1) (Q70E73)	Belongs to the Mig10/Rap1-interacting adaptor molecule/Lamellipodin family of adapter proteins, which function in cell migration. Regulates actin dynamics by direct binding to filamentous actin to	-1.8 (0.047)	High RAPH1 expression is correlated with aggressive breast cancer phenotypes and provides prognostic

		regulate actin network assembly (cytoskelton, plasma membrane)		value in invasive breast cancer [80]
20.	**Serine/threonine-protein kinase** (Nek7)(Q8TDX7)	A kinase that phosphorylates serine or threonine, and plays a role in mitotic cell cycle progression (nucleus)	-1.85 (0.0182)	Oncogenic protein supports mitosis [81]
21.	**Amyloid beta A4 precursor protein-binding family B member 1-interacting protein** (APBB1IP) (Q7Z5R6)	Signal transduction from Ras activation to actin cytoskeletal remodeling (cytoskeleton, cell membrane)	-1.85 (0.00252)	Not reported
22.	**Epidermal growth factor receptor** (EGFR) (P00533)	Receptor tyrosine kinase that binds ligands of the EGF family, activating several signaling cascades to convert extracellular cues into appropriate cellular responses (nucleus, ER, Golgi, endosome, cell membrane)	-1.93 (0.0222)	A key factor in epithelial malignancies, and its activity enhances tumor growth, invasion, and metastasis [82]
23.	**Elongation factor 1-alpha 2** (EEF1A2) (Q05639)	Nucleotide binding that is responsible for the enzymatic delivery of aminoacyl tRNAs to the ribosome (nucleus)	-1.93 (0.0895)	A putative oncogene of lung cancer [83]
24.	**Annexin A3** (ANXA3) (P12429)	Inhibitor of phospholipase A2 that also possesses anti-coagulant properties (cytosol, plasma membrane)	-2.03 (0.0457)	Overexpression promotes proliferation and metastasis in lung, breast, liver, and ovarian carcinomas [84]
25.	**Ras-related protein Rab-27B** (RAB27B) (O00194)	Small GTPase cycles between active GTP-bound and inactive GDP-bound states, and regulates homeostasis of the late endocytic pathway, including endosomal positioning, maturation, and secretion (endosome)	-2.14 (0.00415)	Control exosome secretion in cervical, breast, melanoma, bladder and lung cancers. Promote cell proliferation, cell invasion, and increase chemoresistance of cancer [85]
26.	**Transmembrane protein 120A** (TMEM120A) (*Q9BXJ8*)	Fat cell differentiation, necessary for efficient adipogenesis (nucleus)	-2.19 (0.00425)	Not reported
27.	**Ras GTPase-activating protein nGAP** (RASAL2) (*Q9UJF2*)	Inhibitory regulator of the Ras-cyclic AMP pathway (cytosol)	-2.32 (0.0266)	Overexpressed and promotes tumor progression in CRC [86]
28.	**Catenin beta-1** (CTNNB1) (*P35222*)	Key downstream component of the canonical Wnt signaling pathway (cytoplasm, cell membrane, nucleus)	-2.37 (0.00929)	Potential therapeutic target in cancer [87]
29.	**Protein THEM6** (THEM6) (Q8WUY1)	Mesenchymal stem cell protein (secretory)	-2.39 (0.0489)	Not reported
30.	**Protein S100-A6** (S100-A6) (P06703)	Calcium signaling regulation that may function in stimulating Ca^{2+}-dependent insulin release, prolactin secretion, and exocytosis (nucleus, cytoplasm, cell membrane)	-2.5 (0.0422)	Overexpressed in several type of cancers such as pancreatic [88] and gastric cancer [89] and promotes tumerogenesis
31.	**Pituitary tumor-transforming gene 1 protein-interacting**	Negative regulation of DNA damage response (nucleus, cytoplasm)	-2.92 (0.00117)	Methylation level of its promoter region may be a biomarker for early

	**protein** (PTTG1IP) (*P53801*)			diagnosis of lung cancer [90]
32.	**TGF-beta receptor type-2** (TGFBR2) (*P37173*)	Membranal serine/threonine kinase that binds TGF-beta and the receptor/ligand complex phosphorylate proteins, which then enter the nucleus and regulate the transcription of a subset of genes related to cell proliferation (cell membrane)	-3.17 (0.049)	Expression in cancer-associated fibroblasts regulates breast cancer cell growth and survival and is a prognostic marker in pre-menopausal breast cancer [91]
33.	**Protein S100-A16** (S100-A16) (Q96FQ6)	Calcium-binding protein that may function in protein homo-dimerization activity (nucleus, cytoplasm)	-3.44 (0.0279)	Overexpressed and associated with the prognosis of CRC patients and is a potential biomarker for CRC [92]
34.	**Brain-specific angiogenesis inhibitor 1-associated protein 2** (BAIAP2) (Q9UQB8)	Adapter protein that links membrane-bound small G-proteins to cytoplasmic effector proteins (cytoplasm)	-4.33 (0.00727)	Not reported
35.	**Transmembrane 4 L6 family member 1** (TM4SF1) (*P30408*)	Mediate signal transduction events that play a role in the regulation of cell development, activation, growth and motility (cell membrane)	-4.87 (0.00777)	Promotes non-small cell lung cancer proliferation, invasion, and chemo-resistance [93]
36.	**Syntaxin-3** (STX3) (*Q13277*)	Potentially involved in docking of synaptic vesicles at presynaptic active zones (cell membrane)	-4.95 (0.0152)	Correlates with poor prognosis and prognostic factors of lung adenocarcinoma [94]
37.	**Prospero homeobox protein 1** (PROX1) (*Q92786*)	Transcription factor involved in developmental processes (nucleus)	-5.0 (0.0785)	Overexpressed in lung cancer and is a therapeutic target [95]
38.	**Protein S100-A3** (S100-A3) (P33764)	Calcium and zinc binding protein involved in the regulation of a number of cellular processes such as cell cycle progression and differentiation (cytoplasm)	-5.11 (0.0273)	A novel target for lung cancer and acute myeloid leukemia [96]
39.	**Cyclin-dependent kinase 6** (CDK6) (*Q00534*)	Serine/threonine-protein kinase involved in the control of the cell cycle and differentiation (cytoplasm, nucleus)	-5.42 (0.0542)	Upregulated CDK6 activity is associated with cancer development and is an established drug target [97]
40.	**Sushi domain-containing protein 2** (SUSD2) (*Q9UGT4*)	Negative regulation of cell cycle G1/S phase transition (cell membrane)	-6.43 (0.00572)	Promotes cancer metastasis and confers cisplatin resistance in high-grade ovarian cancer [98]
41.	**ADP-ribosyl cyclase/cyclic ADP-ribose hydrolase 1** (CD38) (*P28907*)	Synthesizes the second messenger cyclic ADP-ribose and nicotinate-adenine dinucleotide phosphate (cell membrane)	-7.54 (0.0429)	Mediates immunosuppression, which facilitates tumor cell escape from the PD-1/PD-l1 blockade in lung cancer [99]

Table S8. Alterations in the expression of immune response-related proteins in SMAC-KO A549 human cancer cells

LC-HR MS/MS analysis was performed as described in the Materials and Methods section. Differentially expressed proteins (*p*-value <0.05, FC |1.5|) were filtered, and those proteins differentially expressing SMAC-KO cells are presented. For each protein, the name, fold change (FC) and *p*-value, and its function, subcellular localization, and relevance to cancer are indicated.

No.	Protein name (UniProtKB)	Proposed function (Cell localization)	FC (*p*-value)	Relation to cancer
1.	**CD70 antigen (**CD70) **(P32970)**	Co-stimulatory receptor belonging to the TNF family that is expressed on naive T and B cells and on NK cells, and is the ligand for CD27 (cell membrane)	+2.02 (0.00803)	Overexpressed in most osteosarcomas [100]
2.	**Cyclic GMP, AMP synthase (**CGAS) (Q8N884)	Nucleotidyl-transferase that catalyzes the formation of cyclic GMP and AMP, and plays a key role in innate immunity (nucleus, cytoplasm, cell membrane)	+1.79 (0.00903)	Dichotomous roles in tumor immunity and development [101]
3.	**Transmembrane protein 179B** (TMEM179B) (Q7Z7N9)	Neutrophil degranulation that regulates exocytosis of secretory granules containing preformed mediators such as proteases, lipases, and inflammatory mediators by a neutrophil (plasma membrane)	-2.2 (0.004851)	Not reported
4.	**Tetraspanin-4** (TSPAN4) (O14817)	Tetraspanins can influence cell adhesion, migration, invasion, signaling, cell–cell fusion, and survival. Interacts with various immune receptors, forming multimolecular complexes that can initiate immune cell signaling [102] (cell membrane)	-2.76 (0.0331)	Promotes multiple cancer stages [103]
5.	**Complement decay-accelerating factor** (CD55) (P08174)	Complement system cascade protein that plays an anti-adhesive role in human neutrophil transmigration across the mucosal epithelia [104]. Its overexpression in tumors results in immune escape adopted to avoid recognition by the immune system or survival from antibody-mediated immunotherapy (cell membrane)	-4.96 (0.0216)	Known to promote several types of cancer and its inhibitors are being used for targeted cancer therapy [105]. Protects human tumor cells from complement-mediated cytotoxicity [106, 107]
6.	**Aldehyde dehydrogenase family 3 member B1 (**ALDH3B1) (P43353)	Oxidizes medium- and long-chain saturated and unsaturated aldehydes. It is proposed to play a significant role in the tumor immune landscape by modulating immunocytes [108] (cell membrane)	-1.78 (0.0105)	Stem cell marker in solid tumors [109]
7.	**Cathepsin D** (CTSD) (*P07339*)	A lysosomal acid protease found in neutrophils and monocytes that regulates the infiltration of neutrophils (Lysosome)	-2.1 (0.0294)	Biomarker for osteosarcomas, pulmonary metastases, and other bone malignancies [110], and promotes invasion in salivary adenoid cystic carcinoma and breast cancer[111, 112]
8.	**Transmembrane protein 179B** (TMEM179B) (*Q7Z7N9*)	Involved in neutrophil degranulation (cell membrane)	-2.2 (0.00851	Not reported
9.	**Ferritin light chain** (FTL) (P02792)	A subunit of ferritin that is the main form of iron storage protein. A potent direct chemotactic for neutrophil modulation	-4.97 (0.0271)	Involved in tumor-associated macrophages and T-regulatory cell infiltration in most solid

		known to influence tumor immunity [113] (cytoplasm, lysosomes)		tumors [113, 114]. Promotes EMT and chemoresistance in glioma [115] and CRC[115]. Elevated in glioblastoma, its silencing inhibits glioblastoma cell proliferation [116]

Table S9. Alterations in the expression of DNA and RNA-related proteins in SMAC-KO A549 human cancer cells

LC-HR MS/MS analysis was performed as described in the Materials and Methods section. Differentially expressed proteins (*p*-value <0.05, FC |1.5|) were filtered, and those proteins differentially expressing SMAC-KO cells are presented. For each protein, the name, fold change (FC), and *p*-value, as well as its function, subcellular localization, and relevance to cancer are indicated.

No.	Protein name (UniProtKB)	Proposed function (Cellular localization)	FC (*p*-value)	Relation to cancer
1.	**Schlafen family member 11** (SLFN11) (*Q7Z7L1*)	Inhibitor of DNA replication that promotes cell death in response to DNA damage (nucleus)	+14.5 (0.0000572)	Overexpressed in ovarian and colorectal adenocarcinomas [117]
2.	**Integrator complex subunit 9** (INTS9) (*Q9NV88*)	Component of the integrator complex that is involved in small nuclear RNA U1 and U2 transcription and in their 3’-box-dependent processing. Mediates recruitment of dynein to the nuclear envelope (nucleus)	+2.88 (0.00959)	Not reported
3.	**Protein CASC3** (CASC3) (*O15234*)	Core component of the splicing-dependent multiprotein exon junction complex deposited at splice junctions on mRNAs (nucleus)	+2.13 (0.013)	Not reported
4.	**Insulin-like growth factor 2 mRNA-binding protein 2** (IGF2BP2) (*Q9Y6M1*)	RNA-binding factor that recruits target transcripts to cytoplasmic protein-RNA complexes (nucleus, cytoplasm)	+2.07 (0.00634)	Tumor promoter that drives cancer proliferation [118]
5.	**Telomere-associated protein** (RIF1) (*Q5UIP0*)	Key regulator of TP53BP1 that plays a key role in the repair of double-strand DNA breaks (DSBs) in response to DNA damage (nucleus)	+1.9 (0.0331)	Highly expressed in over 80% of tumors [119]
6.	**Thyroid transcription factor 1-associated protein 26** (CCDC59) (*Q9P031*)	Component of the transcription complexes of the pulmonary surfactant-associated protein-B and -C (nucleus)	+1.88 (0.0425)	Overexpression in lung adenocarcinoma [120]
7.	**Serine/arginine-rich splicing factor 9** (SRSF9) (*Q13242*)	A member of the serine/arginine (SR)-rich family of pre-mRNA splicing factors, which constitute part of the spliceosome (nucleus)	+1.76 (0.0238)	Up-regulated in bladder cancer and therapeutic target [121]
8.	**DNA-directed RNA polymerase I subunit** (RPA2) (*Q9H9Y6*)	Responsible for the polymerization of ribonucleotides into a sequence complementary to the DNA (nucleus)	+1.76 (0.00922)	Not reported
9.	**Protein** (SCAF11) (*Q99590*)	Plays a role in pre-mRNA alternative splicing by regulating the spliceosome assembly (nucleus)	+1.75 (0.0142)	Not reported

10.	**REST co-repressor 1** (RCOR1) (*Q9UKL0*)	Essential component of the BRAF-HDAC complex that is a co-repressor of the transcription of neuron-specific genes in non-neuronal cells (nucleus)	+1.71 (0.0198)	Knocking-down REST expression in breast cancer cells increases proliferation, suppress apoptosis, reduced sensitivity to anticancer drug [122]
11.	**AT-rich interactive domain-containing protein 2** (ARID2) (*Q68CP9*)	Involved in transcriptional activation and repression of select genes by chromatin remodeling (nucleus)	+1.70 (0.0119)	Suppresses tumor cell growth via repression of cyclin D1 and cyclin E1 expression, thereby retarding cell proliferation in hepatoma cells [123]
12.	**RNA polymerase-associated protein** (LEO1) (*Q8WVC*0)	Component of transcription by RNA polymerase II, involved in regulation of development and maintenance of embryonic stem cell pluripotency (nucleus)	+1.62 (0.0252)	Not reported
13.	**Probable ATP-dependent RNA helicase** (DDX47) (*Q9H0S4*)	Involved in apoptosis, and possibly function r in rRNA processing and mRNA splicing. Associates with pre-rRNA precursors (nucleus)	+1.59 (0.0329)	Not reported
14.	**Protein bicaudal C homolog 1** (BICC1) (*Q9H69*4)	RNA-binding protein that acts as a negative regulator of Wnt signaling. May be involved in regulating gene expression during embryonic development (cytoplasm)	-2.32 (0.00673)	Upregulated in oral cancer tissues and cell line and increases proliferation [124]

Table S10. Alterations in the expression of protein synthesis- and degradation-related proteins in SMAC-KO A549 human cancer cells

LC-HR MS/MS analysis was performed as described in the Materials and Methods section. Differentially expressed proteins (*p*-value <0.05, FC |1.5|) were filtered, and those proteins differentially expressing SMAC-KO cells are presented. For each protein, the name, fold change (FC), and *p*-value, as well as its function, subcellular localization, and relevance to cancer are indicated.

No.	Protein name (UniProtKB)	Proposed function (Cellular localization)	FC (*p*-value)	Relation to cancer
1.	**Eukaryotic translation initiation factor 1A, Y-chromosomal** (EIF1AY) (*O14602*)	Required for maximal protein biosynthesis. Enhances ribosome dissociation into subunits and stabilizes the binding of the initiator Met-tRNA(I) to 40 S ribosomal subunits (nucleus)	+5.94 (0.000295)	Its unstructured N-terminal tail (NTT) is frequently mutated in several malignancies [125]
2.	**rRNA-processing protein FCF1 homolog** (FCF1) (*Q9Y324*)	Essential protein involved in pre-rRNA processing and 40S ribosomal subunit assembly (nucleolus)	+3.18 (0.0757)	Not reported
3.	**Legumain LGMN** (Q99538)	Hydrolyzes asparaginyl bonds. Required for normal lysosomal protein degradation in renal proximal tubules and degradation of internalized EGFR, thereby regulating cell proliferation (lysosome)	+3.11 (0.000782)	Overexpressed in cancer [126, 127]
4.	**60S ribosomal protein L22-like 1** (RPL22L1) (*Q6P5R6*)	Essential components of the ribosome, may be critical for both itscregulatory and structural functions (ribosome)	+2.41 (0.0374)	Highly mutated in various types of human cancers [128]

5.	**Probable E3 ubiquitin-protein ligase** (IRF2BPL) (*Q9H1B7*)	Involved in the proteasome-mediated ubiquitin-dependent degradation of target proteins (nucleus)	+2.13 (0.0303)	Targets β catenin for proteasome degradation in gastric cancer [129]
6.	**F-box/WD repeat-containing protein 11** (FBXW11) (*Q9UKB1*)	Ubiquitin-protein ligase complex that mediates the ubiquitination and subsequent proteasomal degradation of target proteins (nucleus, cytoplasm)	+1.8 (0.00997)	Dysregulation of F-box protein-mediated proteolysis leads to malignancies [130]
7.	**Tripartite motif-containing protein 16** (TRIM16) (*O95361*)	E3 ubiquitin ligase that plays an essential role in the organization of the autophagic response and ubiquitination upon lysosomal and phagosomal damage (cytoplasm)	+1.76 (0.0123)	Contributes to cancer and is a potential target in cancer therapy [131]
8.	**Ubiquitin-conjugating enzyme E2 G1** (UBE2G1) (*P62253*)	Accepts ubiquitin from the E1 complex and catalyzes its covalent attachment to other proteins (cytosol)	+1.71 (0.0209)	Not reported
9.	**Ubiquitin-conjugating enzyme E2 E1** (UBE2E1) (P51965)	Accepts ubiquitin from the E1 complex and catalyzes its covalent attachment to other proteins. Mediates the selective degradation of short-lived and abnormal proteins (nucleus)	+1.64 (0.0202)	Dysregulated in a variety of cancers. Involved in various tumor-promoting processes including DNA repair, cell cycle progression, apoptosis, and oncogenic signaling [132]
10.	**Zinc finger CCCH domain-containing protein 15** (ZC3H15) (*Q8WU90*)	Protects DRG1 from proteolytic degradation and stimulates its GTPase activity (nucleus, cytoplasm)	+1.64 (0.0422)	Not reported
11.	**28S ribosomal protein S25** (MRPS25) (*P82663*)	A ribosomal protein, a component of the 40S subunit that belongs to the S25E family of ribosomal proteins and likely plays a role in general protein synthesis (mitochondria)	-1.53 (0.0246)	Overexpressed in breast cancer stem cells [133]
12.	**Proteasome subunit beta type-9** (PSMB9) (*P28065*)	Proteasomal degradation pathway protein, involved in antigen processing to generate class I binding peptides (nucleus, cytoplasm)	-1.55 (0.032)	Overexpressed in AML, breast, myeloid, and lymphoid cancer cells, liver, bladder, colon, and uterine cancers [134]
13.	**Mitochondrial ribosome-associated GTPase 1** (MTG1) (*Q9BT17*)	Regulates mitochondrial ribosome assembly and translational activity (mitochondria)	-1.79 (0.0102)	High ranked cancer-association [135]
14.	**COMM domain-containing protein 9** (COMMD9) (*Q9P000*)	May modulate activity of cullin-RING E3 ubiquitin ligase (CRL) complexes (nucleus, cytoplasm)	-2.16 (0.00899)	Promotes the development of non-small cell lung cancer [136]
15.	**Cystatin-B** (CSTB) (P04080)	Intracellular thiol proteinase inhibitor. Reversible inhibitor of cathepsins L, H and B (nucleus, cytoplasm)	-2.27 (0.00468)	Overexpressed and a biomarker for ovarian [137], breast [138], and hepatocellular carcinomas [139]
16.	**Protein** (IMPACT) (Q9P2X3)	Translational regulator that ensures constant high levels of translation upon a variety of stress conditions, such as amino acid starvation, UV-C irradiation, and glucose deprivation (cytoplasm)	-3.06 (0.00577)	Not reported

17.	**Selenocysteine-specific elongation factor** (EEFSEC) (*P57772*)	Translation factor necessary for the incorporation of selenocysteine into proteins (cytoplasm, nucleus)	-5.45 (0.0165)	Not reported
18.	**FUN14 domain-containing protein 2** (FUNDC2) (Q9BWH2)	Play a role in mitophagy, interacts with molecules like LC3B, which is found on MAM, to maintain good mitochondrial quality by forming mitophagosomes (mitochondria, nucleus)	-5.77 (0.00106)	Not reported
19.	**Glutathione peroxidase 1** (GPX1) (P07203)	Antioxidant enzyme that enzymatically reduces hydrogen peroxide, and essential for growth factor-mediated signal transduction, mitochondrial function, and maintenance of normal thiol redox-balance. (cytoplasm)	-6.35 (0.00139)	Upregulated in several major cancer types such as of the lungs and breast, and promotes chemoresistance [140]

Table S11. Alterations in the expression of human proteins involved in epigenetics in SMAC-KO A549 human cancer cells

LC-HR MS/MS analysis was performed as described in the Materials and Methods section. Differentially expressed proteins (*p*-value <0.05, FC |1.5|) were filtered, and those proteins differentially expressing SMAC-KO cells are presented. For each protein, the name, fold change (FC), and *p*-value, as well as its function, subcellular localization, and relevance to cancer are indicated.

No.	Protein name (UniProtKB)	Proposed function (Cellular localization)	FC (*p*-value)	Relation to cancer
1.	**Histone H1.5** (H1-5) (P16401)	Regulator of individual gene transcription through chromatin remodeling, nucleosome spacing, and DNA methylation (nucleus)	+12.2 (0.000294)	Overexpression in prostate adenocarcinomas [141]
2.	**Histone H3.2** (H3-2) (Q71DI3)	Core component of nucleosome (nucleus)	+2.28 (0.0103)	Mutated in cancer [142]
3.	**Disco-interacting protein 2 homolog B** (DIP2B) (Q9P265)	DNA methylation (nucleus, cytoplasm, cell membrane)	+1.82 (0.0133)	Not reported
4.	**Chromobox protein homolog 5** (P45973) (CBX5)	Component of heterochromatin that recognizes and binds histone H3 tails methylated at 'Lys-9' (H3K9me), leading to epigenetic repression (nucleus)	+1.76 (0.0188)	Downregulated in metastatic breast cancer compared to non-metastatic cancer [143]
5.	**Bromodomain adjacent to zinc finger domain protein 1A** (BAZ1A) (Q9NRL2)	Plays an important role in chromatin remodeling (nucleus)	+1.65 (0.0379)	Not reported
6.	**SWI/SNF complex subunit** (SMARCC2) (Q8TAQ2)	Involved in transcriptional activation and repression of select genes by chromatin remodeling (nucleus)	+1.58 (0.0225)	Highly mutated in gastric and colorectal cancers [144]
7.	**Protein FRG1** (FRG1) (Q14331)	RNA splicing and epigenetic modulation (nucleus, cytoplasm)	+1.57 (0.0227)	Reduced expression promotes prostate cancer progression and affects cell migration and invasion [145]

8.	**Periphilin-1** (PPHLN1) (Q8NEY8)	Component of the HUSH, a multiprotein complex that mediates epigenetic repression (nucleus, cytoplasm)	+1.55 (0.033)	Not reported
9.	**Histone H2A type 1-C** (H2AC6) (Q93077)	Core component of nucleosome (nucleus)	-1.77 (0.0199)	Not reported
10.	**NAD-dependent protein deacetylase sirtuin-2** (SIRT2) (Q8IXJ6)	NAD-dependent deacetylates internal lysines on histone and alpha-tubulin, as well as key transcription factors (cytoplasm, nucleus, plasma membrane)	-2.31 (0.0116)	Acts as both an oncogene and a tumor suppressor [146]
11.	**CXXC-type zinc finger protein 1** (CXXC1) (Q9P0U4)	Transcriptional activator, exhibiting a unique DNA binding specificity for CpG unmethylated motifs with a preference for CpGG, (nucleus)	-135 (5.18E-6)	Not reported

References

1. Konson, A., et al., *Pigment epithelium-derived factor and its phosphomimetic mutant induce JNK-dependent apoptosis and p38-mediated migration arrest.* J Biol Chem, 2011. **286**(5): p. 3540-51.
2. Shah, T., et al., *Molecular causes of elevated phosphoethanolamine in breast and pancreatic cancer cells.* NMR Biomed, 2018. **31**(8): p. e3936.
3. Zhang, X., et al., *Phospholipid Phosphatase 4 promotes proliferation and tumorigenesis, and activates Ca(2+)-permeable Cationic Channel in lung carcinoma cells.* Mol Cancer, 2017. **16**(1): p. 147.
4. Kurabe, N., et al., *Accumulated phosphatidylcholine (16:0/16:1) in human colorectal cancer; possible involvement of LPCAT4.* Cancer Sci, 2013. **104**(10): p. 1295-302.
5. Vaclavikova, R., D.J. Hughes, and P. Soucek, *Microsomal epoxide hydrolase 1 (EPHX1): Gene, structure, function, and role in human disease.* Gene, 2015. **571**(1): p. 1-8.
6. Schug, Z.T., et al., *Acetyl-CoA synthetase 2 promotes acetate utilization and maintains cancer cell growth under metabolic stress.* Cancer Cell, 2015. **27**(1): p. 57-71.
7. Wang, H.P., et al., *Phospholipid hydroperoxide glutathione peroxidase protects against singlet oxygen-induced cell damage of photodynamic therapy.* Free Radic Biol Med, 2001. **30**(8): p. 825-35.
8. Nomura, D.K., et al., *Monoacylglycerol lipase regulates a fatty acid network that promotes cancer pathogenesis.* Cell, 2010. **140**(1): p. 49-61.
9. Nagpal, J.K., et al., *Profiling the expression pattern of GPI transamidase complex subunits in human cancer.* Mod Pathol, 2008. **21**(8): p. 979-91.
10. Paul, A., et al., *A New Role for the Mitochondrial Pro-apoptotic Protein SMAC/Diablo in Phospholipid Synthesis Associated with Tumorigenesis.* Mol Ther, 2018. **26**(3): p. 680-694.
11. Pimiento, J.M., et al., *Annexin A8 Is a Prognostic Marker and Potential Therapeutic Target for Pancreatic Cancer.* Pancreas, 2015. **44**(1): p. 122-7.
12. Xie, J., et al., *Solute carrier transporters: potential targets for digestive system neoplasms.* Cancer Manag Res, 2018. **10**: p. 153-166.
13. Detchokul, S., et al., *Tetraspanins as regulators of the tumour microenvironment: implications for metastasis and therapeutic strategies.* Br J Pharmacol, 2014. **171**(24): p. 5462-90.
14. Yang, Z., et al., *Adenine nucleotide (ADP/ATP) translocase 3 participates in the tumor necrosis factor induced apoptosis of MCF-7 cells.* Mol Biol Cell, 2007. **18**(11): p. 4681-9.
15. Zhi, J., et al., *Support vector machine classifier for prediction of the metastasis of colorectal cancer.* Int J Mol Med, 2018. **41**(3): p. 1419-1426.
16. Antonacopoulou, A.G., et al., *POLR2F, ATP6V0A1 and PRNP expression in colorectal cancer: new molecules with prognostic significance?* Anticancer Res, 2008. **28**(2B): p. 1221-7.
17. Wang, X., et al., *Reduced expression of tocopherol-associated protein (TAP/Sec14L2) in human breast cancer.* Cancer Invest, 2009. **27**(10): p. 971-7.
18. Kim, E., et al., *Upregulation of SLC2A3 gene and prognosis in colorectal carcinoma: analysis of TCGA data.* BMC Cancer, 2019. **19**(1): p. 302.

19. Ahn, W.S., et al., *Searching for pathogenic gene functions to cervical cancer.* Gynecol Oncol, 2004. **93**(1): p. 41-8.
20. Du, X., et al., *Akt activation increases cellular cholesterol by promoting the proteasomal degradation of Niemann-Pick C1.* Biochem J, 2015. **471**(2): p. 243-53.
21. Zhang, J., et al., *VPS52 induces apoptosis via cathepsin D in gastric cancer.* J Mol Med (Berl), 2017. **95**(10): p. 1107-1116.
22. Bie, Y. and Z. Zhang, *RAB8A a new biomarker for endometrial cancer?* World J Surg Oncol, 2014. **12**: p. 371.
23. Vainio, P., et al., *High-throughput transcriptomic and RNAi analysis identifies AIM1, ERGIC1, TMED3 and TPX2 as potential drug targets in prostate cancer.* PLoS One, 2012. **7**(6): p. e39801.
24. Liu, J., et al., *Syntenin1/MDA-9 (SDCBP) induces immune evasion in triple-negative breast cancer by upregulating PD-L1.* Breast Cancer Res Treat, 2018. **171**(2): p. 345-357.
25. Cui, L., et al., *Syntenin-1 is a promoter and prognostic marker of head and neck squamous cell carcinoma invasion and metastasis.* Oncotarget, 2016. **7**(50): p. 82634-82647.
26. Cao, W.F., et al., *[Relationship between SLP-2 expression and prognosis in laryngeal squamous cell carcinoma and mammary invasive carcinoma].* Zhonghua Bing Li Xue Za Zhi, 2010. **39**(5): p. 332-7.
27. Hirota, J., et al., *Carbonic anhydrase-related protein is a novel binding protein for inositol 1,4,5-trisphosphate receptor type 1.* Biochem J, 2003. **372**(Pt 2): p. 435-41.
28. Lu, S.H., et al., *Effect of carbonic anhydrase-related protein VIII expression on lung adenocarcinoma cell growth.* Lung Cancer, 2004. **44**(3): p. 273-80.
29. Ma, H.L., et al., *CA8 promotes RCC proliferation and migration though its expression level is lower in tumor compared to adjacent normal tissue.* Biomed Pharmacother, 2020. **121**: p. 109578.
30. Nishikata, M., et al., *Carbonic anhydrase-related protein VIII promotes colon cancer cell growth.* Mol Carcinog, 2007. **46**(3): p. 208-14.
31. Mishra, M.N., K.K. Vangara, and S. Palakurthi, *Transcriptional targeting of human liver carboxylesterase (hCE1m6) and simultaneous expression of anti-BCRP shRNA enhances sensitivity of breast cancer cells to CPT-11.* Anticancer Res, 2014. **34**(11): p. 6345-51.
32. Sun, W.Y., et al., *Evaluation of the Expression of Amine Oxidase Proteins in Breast Cancer.* Int J Mol Sci, 2017. **18**(12).
33. Zhuang, H., et al., *Glycine decarboxylase induces autophagy and is downregulated by miRNA-30d-5p in hepatocellular carcinoma.* Cell Death Dis, 2019. **10**(3): p. 192.
34. Federer-Gsponer, J.R., et al., *Delineation of human prostate cancer evolution identifies chromothripsis as a polyclonal event and FKBP4 as a potential driver of castration resistance.* J Pathol, 2018. **245**(1): p. 74-84.
35. Elliott, W.L. and G. Weber, *Proliferation-linked increase in phosphoribosylformylglycinamidine synthetase activity (EC 6.3.5.3).* Cancer Res, 1984. **44**(6): p. 2430-4.
36. Nishimura, T., et al., *Cancer stem-like properties and gefitinib resistance are dependent on purine synthetic metabolism mediated by the mitochondrial enzyme MTHFD2.* Oncogene, 2019. **38**(14): p. 2464-2481.
37. Penzo, M., et al., *RNA Pseudouridylation in Physiology and Medicine: For Better and for Worse.* Genes (Basel), 2017. **8**(11).
38. Hiyama, N., et al., *Glutamate-cysteine ligase catalytic subunit is associated with cisplatin resistance in lung adenocarcinoma.* Jpn J Clin Oncol, 2018. **48**(4): p. 303-307.
39. Kostourou, V., et al., *Dimethylarginine dimethylaminohydrolase I enhances tumour growth and angiogenesis.* Br J Cancer, 2002. **87**(6): p. 673-80.
40. Hu, H., et al., *Acetylation of PGK1 promotes liver cancer cell proliferation and tumorigenesis.* Hepatology, 2017. **65**(2): p. 515-528.
41. Hussain, M.R., D.C. Hoessli, and M. Fang, *N-acetylgalactosaminyltransferases in cancer.* Oncotarget, 2016. **7**(33): p. 54067-54081.
42. Matsumoto, A., et al., *Aldehyde dehydrogenase 1B1: a novel immunohistological marker for colorectal cancer.* Br J Cancer, 2017. **117**(10): p. 1537-1543.
43. Bartoszek, A. and C.R. Wolf, *Enhancement of doxorubicin toxicity following activation by NADPH cytochrome P450 reductase.* Biochem Pharmacol, 1992. **43**(7): p. 1449-57.
44. Bieche, I., et al., *Variations in the mRNA expression of poly(ADP-ribose) polymerases, poly(ADP-ribose) glycohydrolase and ADP-ribosylhydrolase 3 in breast tumors and impact on clinical outcome.* Int J Cancer, 2013. **133**(12): p. 2791-800.
45. Lai, C.Y., et al., *Identification of UAP1L1 as a critical factor for protein O-GlcNAcylation and cell proliferation in human hepatoma cells.* Oncogene, 2019. **38**(3): p. 317-331.
46. Qin, W., et al., *Research Progress on PARP14 as a Drug Target.* Front Pharmacol, 2019. **10**: p. 172.

47. Huang, H.L., et al., *Argininosuccinate lyase is a potential therapeutic target in breast cancer.* Oncol Rep, 2015. **34**(6): p. 3131-9.
48. Huang, H.L., et al., *Silencing of argininosuccinate lyase inhibits colorectal cancer formation.* Oncol Rep, 2017. **37**(1): p. 163-170.
49. Grant, D.J., et al., *UDP-glucuronosyltransferases and biochemical recurrence in prostate cancer progression.* BMC Cancer, 2017. **17**(1): p. 463.
50. Gopal, S., et al., *Fibronectin-guided migration of carcinoma collectives.* Nat Commun, 2017. **8**: p. 14105.
51. Xiao, J., et al., *Expression of fibronectin in esophageal squamous cell carcinoma and its role in migration.* BMC Cancer, 2018. **18**(1): p. 976.
52. Zeng, X.T., et al., *The clinical significance of COL5A2 in patients with bladder cancer: A retrospective analysis of bladder cancer gene expression data.* Medicine (Baltimore), 2018. **97**(10): p. e0091.
53. Kong, R., et al., *Myo9b is a key player in SLIT/ROBO-mediated lung tumor suppression.* J Clin Invest, 2015. **125**(12): p. 4407-20.
54. Blum, C., et al., *The expression ratio of Map7/B2M is prognostic for survival in patients with stage II colon cancer.* Int J Oncol, 2008. **33**(3): p. 579-84.
55. Fowler, L., et al., *Redistribution and enhanced protein kinase C-mediated phosphorylation of alpha- and gamma-adducin during renal tumor progression.* Cell Growth Differ, 1998. **9**(5): p. 405-13.
56. Chen, G., et al., *p53 target miR-29c-3p suppresses colon cancer cell invasion and migration through inhibition of PHLDB2.* Biochem Biophys Res Commun, 2017. **487**(1): p. 90-95.
57. Miao, Z., et al., *Microtubule actin cross-linking factor 1, a novel potential target in cancer.* Cancer Sci, 2017. **108**(10): p. 1953-1958.
58. Marzinke, M.A., et al., *Calmin expression in embryos and the adult brain, and its regulation by all-trans retinoic acid.* Dev Dyn, 2010. **239**(2): p. 610-9.
59. Zhao, H., et al., *Cell fate regulation by reticulon-4 in human prostate cancers.* J Cell Physiol, 2019. **234**(7): p. 10372-10385.
60. Nakazawa, N., et al., *High Stromal TGFBI in Lung Cancer and Intratumoral CD8-Positive T Cells were Associated with Poor Prognosis and Therapeutic Resistance to Immune Checkpoint Inhibitors.* Ann Surg Oncol, 2020. **27**(3): p. 933-942.
61. Paner, G.P., et al., *Diagnostic utility of antibody to smoothelin in the distinction of muscularis propria from muscularis mucosae of the urinary bladder: a potential ancillary tool in the pathologic staging of invasive urothelial carcinoma.* Am J Surg Pathol, 2009. **33**(1): p. 91-8.
62. Hichino, A., et al., *Down-regulation of Claudin-2 Expression and Proliferation by Epigenetic Inhibitors in Human Lung Adenocarcinoma A549 Cells.* J Biol Chem, 2017. **292**(6): p. 2411-2421.
63. Mezheyeuski, A., et al., *Treatment-related survival associations of claudin-2 expression in fibroblasts of colorectal cancer.* Virchows Arch, 2018. **472**(3): p. 395-405.
64. Tabaries, S., et al., *Afadin cooperates with Claudin-2 to promote breast cancer metastasis.* Genes Dev, 2019. **33**(3-4): p. 180-193.
65. Arimoto, K., et al., *Plakophilin-2 promotes tumor development by enhancing ligand-dependent and -independent epidermal growth factor receptor dimerization and activation.* Mol Cell Biol, 2014. **34**(20): p. 3843-54.
66. Ai, L., et al., *The transglutaminase 2 gene (TGM2), a potential molecular marker for chemotherapeutic drug sensitivity, is epigenetically silenced in breast cancer.* Carcinogenesis, 2008. **29**(3): p. 510-8.
67. Huang, K.Y. and H.H. Lin, *The Activation and Signaling Mechanisms of GPR56/ADGRG1 in Melanoma Cell.* Front Oncol, 2018. **8**: p. 304.
68. Muharram, G., et al., *Tensin-4-dependent MET stabilization is essential for survival and proliferation in carcinoma cells.* Dev Cell, 2014. **29**(4): p. 421-36.
69. Langelotz, C., et al., *Expression of high-mobility-group-protein HMGI-C mRNA in the peripheral blood is an independent poor prognostic indicator for survival in metastatic breast cancer.* Br J Cancer, 2003. **88**(9): p. 1406-10.
70. Vazquez-Cedeira, M. and P.A. Lazo, *Human VRK2 (vaccinia-related kinase 2) modulates tumor cell invasion by hyperactivation of NFAT1 and expression of cyclooxygenase-2.* J Biol Chem, 2012. **287**(51): p. 42739-50.
71. Zhang, Q., et al., *The oncogenic role of Yin Yang 1.* Crit Rev Oncog, 2011. **16**(3-4): p. 163-97.
72. Di Giorgio, E., W.W. Hancock, and C. Brancolini, *MEF2 and the tumorigenic process, hic sunt leones.* Biochim Biophys Acta Rev Cancer, 2018. **1870**(2): p. 261-273.
73. Park, M.H. and J.T. Hong, *Roles of NF-kappaB in Cancer and Inflammatory Diseases and Their Therapeutic Approaches.* Cells, 2016. **5**(2).

74. He, L., et al., *Overexpression of zinc finger protein 384 (ZNF 384), a poor prognostic predictor, promotes cell growth by upregulating the expression of Cyclin D1 in Hepatocellular carcinoma.* Cell Death Dis, 2019. **10**(6): p. 444.
75. Lu, X., et al., *The role of integrins in cancer and the development of anti-integrin therapeutic agents for cancer therapy.* Perspect Medicin Chem, 2008. **2**: p. 57-73.
76. Chen, W., et al., *WNK1 kinase and its partners Akt, SGK1 and NBC-family Na(+)/HCO3(-) cotransporters are potential therapeutic targets for glioblastoma stem-like cells linked to Bisacodyl signaling.* Oncotarget, 2018. **9**(43): p. 27197-27219.
77. Shen, W.G., et al., *Melanoma-associated antigen family protein-D1 regulation of tumor cell migration, adhesion to endothelium, and actin structures reorganization in response to hypoxic stress.* Cell Commun Adhes, 2007. **14**(1): p. 21-31.
78. Huang, T., et al., *SRGAP1, a crucial target of miR-340 and miR-124, functions as a potential oncogene in gastric tumorigenesis.* Oncogene, 2018. **37**(9): p. 1159-1174.
79. Tzeng, H.T. and Y.C. Wang, *Rab-mediated vesicle trafficking in cancer.* J Biomed Sci, 2016. **23**(1): p. 70.
80. Kurozumi, S., et al., *Clinicopathological and prognostic significance of Ras association and pleckstrin homology domains 1 (RAPH1) in breast cancer.* Breast Cancer Res Treat, 2018. **172**(1): p. 61-68.
81. Yissachar, N., et al., *Nek7 kinase is enriched at the centrosome, and is required for proper spindle assembly and mitotic progression.* FEBS Lett, 2006. **580**(27): p. 6489-95.
82. Sasaki, T., K. Hiroki, and Y. Yamashita, *The role of epidermal growth factor receptor in cancer metastasis and microenvironment.* Biomed Res Int, 2013. **2013**: p. 546318.
83. Kawamura, M., et al., *The prognostic significance of eukaryotic elongation factor 1 alpha-2 in non-small cell lung cancer.* Anticancer Res, 2014. **34**(2): p. 651-8.
84. Du, R., et al., *Downregulation of annexin A3 inhibits tumor metastasis and decreases drug resistance in breast cancer.* Cell Death Dis, 2018. **9**(2): p. 126.
85. Li, Z., et al., *Functional implications of Rab27 GTPases in Cancer.* Cell Commun Signal, 2018. **16**(1): p. 44.
86. Pan, Y., et al., *RASAL2 promotes tumor progression through LATS2/YAP1 axis of hippo signaling pathway in colorectal cancer.* Mol Cancer, 2018. **17**(1): p. 102.
87. Shang, S., F. Hua, and Z.W. Hu, *The regulation of beta-catenin activity and function in cancer: therapeutic opportunities.* Oncotarget, 2017. **8**(20): p. 33972-33989.
88. Ohuchida, K., et al., *The role of S100A6 in pancreatic cancer development and its clinical implication as a diagnostic marker and therapeutic target.* Clin Cancer Res, 2005. **11**(21): p. 7785-93.
89. Wang, X.H., et al., *S100A6 overexpression is associated with poor prognosis and is epigenetically up-regulated in gastric cancer.* Am J Pathol, 2010. **177**(2): p. 586-97.
90. Tan, X., et al., *Hypermethylation of the PTTG1IP promoter leads to low expression in early-stage non-small cell lung cancer.* Oncol Lett, 2019. **18**(2): p. 1278-1286.
91. Busch, S., et al., *TGF-beta receptor type-2 expression in cancer-associated fibroblasts regulates breast cancer cell growth and survival and is a prognostic marker in pre-menopausal breast cancer.* Oncogene, 2015. **34**(1): p. 27-38.
92. Sun, X., et al., *S100A16 is a prognostic marker for colorectal cancer.* J Surg Oncol, 2018. **117**(2): p. 275-283.
93. Ye, L., et al., *Transmembrane-4 L-six family member-1 (TM4SF1) promotes non-small cell lung cancer proliferation, invasion and chemo-resistance through regulating the DDR1/Akt/ERK-mTOR axis.* Respir Res, 2019. **20**(1): p. 106.
94. Wang, X., et al., *Membrane Location of Syntaxin-Binding Protein 1 Is Correlated with Poor Prognosis of Lung Adenocarcinoma.* Tohoku J Exp Med, 2020. **250**(4): p. 263-270.
95. Zhu, S.H., et al., *Proliferation of small cell lung cancer cell line reduced by knocking-down PROX1 via shRNA in lentivirus.* Anticancer Res, 2013. **33**(8): p. 3169-75.
96. Gianni, M., et al., *S100A3 a partner protein regulating the stability/activity of RARalpha and PML-RARalpha in cellular models of breast/lung cancer and acute myeloid leukemia.* Oncogene, 2019. **38**(14): p. 2482-2500.
97. Tadesse, S., et al., *Targeting CDK6 in cancer: State of the art and new insights.* Cell Cycle, 2015. **14**(20): p. 3220-30.
98. Xu, Y., et al., *SUSD2 promotes cancer metastasis and confers cisplatin resistance in high grade serous ovarian cancer.* Exp Cell Res, 2018. **363**(2): p. 160-170.
99. Chen, L., et al., *CD38-Mediated Immunosuppression as a Mechanism of Tumor Cell Escape from PD-1/PD-L1 Blockade.* Cancer Discov, 2018. **8**(9): p. 1156-1175.
100. Pahl, J.H., et al., *Expression of the immune regulation antigen CD70 in osteosarcoma.* Cancer Cell Int, 2015. **15**: p. 31.
101. Ng, K.W., et al., *cGAS-STING and Cancer: Dichotomous Roles in Tumor Immunity and Development.* Trends Immunol, 2018. **39**(1): p. 44-54.

102. Cabanas, C., M. Yanez-Mo, and A.B. van Spriel, *Editorial: Functional Relevance of Tetraspanins in the Immune System.* Front Immunol, 2019. **10**: p. 1714.
103. Hemler, M.E., *Tetraspanin proteins promote multiple cancer stages.* Nat Rev Cancer, 2014. **14**(1): p. 49-60.
104. Lawrence, D.W., et al., *Antiadhesive role of apical decay-accelerating factor (CD55) in human neutrophil transmigration across mucosal epithelia.* J Exp Med, 2003. **198**(7): p. 999-1010.
105. Dho, S.H., J.C. Lim, and L.K. Kim, *Beyond the Role of CD55 as a Complement Component.* Immune Netw, 2018. **18**(1): p. e11.
106. Cheung, N.K., et al., *Decay-accelerating factor protects human tumor cells from complement-mediated cytotoxicity in vitro.* J Clin Invest, 1988. **81**(4): p. 1122-8.
107. Kolev, M., L. Towner, and R. Donev, *Complement in cancer and cancer immunotherapy.* Arch Immunol Ther Exp (Warsz), 2011. **59**(6): p. 407-19.
108. Bazewicz, C.G., et al., *Aldehyde dehydrogenase in regulatory T-cell development, immunity and cancer.* Immunology, 2019. **156**(1): p. 47-55.
109. Toledo-Guzman, M.E., et al., *ALDH as a Stem Cell Marker in Solid Tumors.* Curr Stem Cell Res Ther, 2019. **14**(5): p. 375-388.
110. Gemoll, T., et al., *Increased cathepsin D protein expression is a biomarker for osteosarcomas, pulmonary metastases and other bone malignancies.* Oncotarget, 2015. **6**(18): p. 16517-26.
111. Ketterer, S., et al., *Cathepsin D deficiency in mammary epithelium transiently stalls breast cancer by interference with mTORC1 signaling.* Nat Commun, 2020. **11**(1): p. 5133.
112. Zhang, M., et al., *Overexpression Cathepsin D Contributes to Perineural Invasion of Salivary Adenoid Cystic Carcinoma.* Front Oncol, 2018. **8**: p. 492.
113. Hu, Z.W., et al., *Comprehensive analysis of ferritin subunits expression and positive correlations with tumor-associated macrophages and T regulatory cells infiltration in most solid tumors.* Aging (Albany NY), 2021. **13**(8): p. 11491-11506.
114. Brown, R.A.M., et al., *Altered Iron Metabolism and Impact in Cancer Biology, Metastasis, and Immunology.* Front Oncol, 2020. **10**: p. 476.
115. Liu, J., et al., *Hypoxia induced ferritin light chain (FTL) promoted epithelia mesenchymal transition and chemoresistance of glioma.* J Exp Clin Cancer Res, 2020. **39**(1): p. 137.
116. Wu, T., et al., *Expression of Ferritin Light Chain (FTL) Is Elevated in Glioblastoma, and FTL Silencing Inhibits Glioblastoma Cell Proliferation via the GADD45/JNK Pathway.* PLoS One, 2016. **11**(2): p. e0149361.
117. Zoppoli, G., et al., *Putative DNA/RNA helicase Schlafen-11 (SLFN11) sensitizes cancer cells to DNA-damaging agents.* Proc Natl Acad Sci U S A, 2012. **109**(37): p. 15030-5.
118. Dai, N., et al., *IGF2 mRNA binding protein-2 is a tumor promoter that drives cancer proliferation through its client mRNAs IGF2 and HMGA1.* Elife, 2017. **6**.
119. Liu, Y.B., et al., *RIF1 promotes human epithelial ovarian cancer growth and progression via activating human telomerase reverse transcriptase expression.* J Exp Clin Cancer Res, 2018. **37**(1): p. 182.
120. Zhao, Q., et al., *Thyroid transcription factor-1 expression is significantly associated with mutations in exon 21 of the epidermal growth factor receptor gene in Chinese patients with lung adenocarcinoma.* Onco Targets Ther, 2015. **8**: p. 2469-78.
121. Yoshino, H., et al., *Tumor suppressive microRNA-1 mediated novel apoptosis pathways through direct inhibition of splicing factor serine/arginine-rich 9 (SRSF9/SRp30c) in bladder cancer.* Biochem Biophys Res Commun, 2012. **417**(1): p. 588-93.
122. Lv, H., et al., *Expression and functions of the repressor element 1 (RE-1)-silencing transcription factor (REST) in breast cancer.* J Cell Biochem, 2010. **110**(4): p. 968-74.
123. Duan, Y., et al., *Chromatin remodeling gene ARID2 targets cyclin D1 and cyclin E1 to suppress hepatoma cell progression.* Oncotarget, 2016. **7**(29): p. 45863-45875.
124. Wang, H., et al., *miR-101-3p and miR-199b-5p promote cell apoptosis in oral cancer by targeting BICC1.* Mol Cell Probes, 2020: p. 101567.
125. Sehrawat, U., et al., *Cancer-Associated Eukaryotic Translation Initiation Factor 1A Mutants Impair Rps3 and Rps10 Binding and Enhance Scanning of Cell Cycle Genes.* Mol Cell Biol, 2019. **39**(3).
126. Liu, C., et al., *Overexpression of legumain in tumors is significant for invasion/metastasis and a candidate enzymatic target for prodrug therapy.* Cancer Res, 2003. **63**(11): p. 2957-64.
127. Mai, C.W., F.F. Chung, and C.O. Leong, *Targeting Legumain As a Novel Therapeutic Strategy in Cancers.* Curr Drug Targets, 2017. **18**(11): p. 1259-1268.
128. Cao, B., et al., *Cancer-mutated ribosome protein L22 (RPL22/eL22) suppresses cancer cell survival by blocking p53-MDM2 circuit.* Oncotarget, 2017. **8**(53): p. 90651-90661.
129. Higashimori, A., et al., *Forkhead Box F2 Suppresses Gastric Cancer through a Novel FOXF2-IRF2BPL-beta-Catenin Signaling Axis.* Cancer Res, 2018. **78**(7): p. 1643-1656.

130. Wang, Z., et al., *Roles of F-box proteins in cancer.* Nat Rev Cancer, 2014. **14**(4): p. 233-47.
131. Mandell, M.A., B. Saha, and T.A. Thompson, *The Tripartite Nexus: Autophagy, Cancer, and Tripartite Motif-Containing Protein Family Members.* Front Pharmacol, 2020. **11**: p. 308.
132. Hosseini, S.M., et al., *E2 ubiquitin-conjugating enzymes in cancer: Implications for immunotherapeutic interventions.* Clin Chim Acta, 2019. **498**: p. 126-134.
133. Lamb, R., et al., *Targeting tumor-initiating cells: eliminating anabolic cancer stem cells with inhibitors of protein synthesis or by mimicking caloric restriction.* Oncotarget, 2015. **6**(7): p. 4585-601.
134. Rouette, A., et al., *Expression of immunoproteasome genes is regulated by cell-intrinsic and -extrinsic factors in human cancers.* Sci Rep, 2016. **6**: p. 34019.
135. Rubio-Viqueira, B., et al., *An in vivo platform for translational drug development in pancreatic cancer.* Clin Cancer Res, 2006. **12**(15): p. 4652-61.
136. Zhan, W., et al., *COMMD9 promotes TFDP1/E2F1 transcriptional activity via interaction with TFDP1 in non-small cell lung cancer.* Cell Signal, 2017. **30**: p. 59-66.
137. Wang, X., et al., *Cystatin B is a progression marker of human epithelial ovarian tumors mediated by the TGF-beta signaling pathway.* Int J Oncol, 2014. **44**(4): p. 1099-106.
138. Butinar, M., et al., *Stefin B deficiency reduces tumor growth via sensitization of tumor cells to oxidative stress in a breast cancer model.* Oncogene, 2014. **33**(26): p. 3392-400.
139. Lee, M.J., et al., *Identification of cystatin B as a potential serum marker in hepatocellular carcinoma.* Clin Cancer Res, 2008. **14**(4): p. 1080-9.
140. Chen, B., et al., *Glutathione Peroxidase 1 Promotes NSCLC Resistance to Cisplatin via ROS-Induced Activation of PI3K/AKT Pathway.* Biomed Res Int, 2019. **2019**: p. 7640547.
141. Khachaturov, V., et al., *Histone H1.5, a novel prostatic cancer marker: an immunohistochemical study.* Hum Pathol, 2014. **45**(10): p. 2115-9.
142. Lowe, B.R., et al., *Histone H3 Mutations: An Updated View of Their Role in Chromatin Deregulation and Cancer.* Cancers (Basel), 2019. **11**(5).
143. Vad-Nielsen, J., et al., *Regulatory dissection of the CBX5 and hnRNPA1 bi-directional promoter in human breast cancer cells reveals novel transcript variants differentially associated with HP1alpha down-regulation in metastatic cells.* BMC Cancer, 2016. **16**: p. 32.
144. Kim, S.S., et al., *Frameshift mutations of a chromatin-remodeling gene SMARCC2 in gastric and colorectal cancers with microsatellite instability.* APMIS, 2013. **121**(2): p. 168-9.
145. Tiwari, A., et al., *Reduced FRG1 expression promotes prostate cancer progression and affects prostate cancer cell migration and invasion.* BMC Cancer, 2019. **19**(1): p. 346.
146. Zhang, L., S. Kim, and X. Ren, *The Clinical Significance of SIRT2 in Malignancies: A Tumor Suppressor or an Oncogene?* Front Oncol, 2020. **10**: p. 1721.
