# Supplementary material for: Non-apoptotic activity of the mitochondrial protein SMAC/Diablo in lung cancer: Novel target to disrupt survival, inflammation, and immunosuppression
Source: Front Oncol. 2022 Sep 14;12:992260. doi: 10.3389/fonc.2022.992260 (PMC9515501; doi:10.3389/fonc.2022.992260)
Supplement: Supplementary file 2 [file Presentation_1.pptx]

## Slide 1
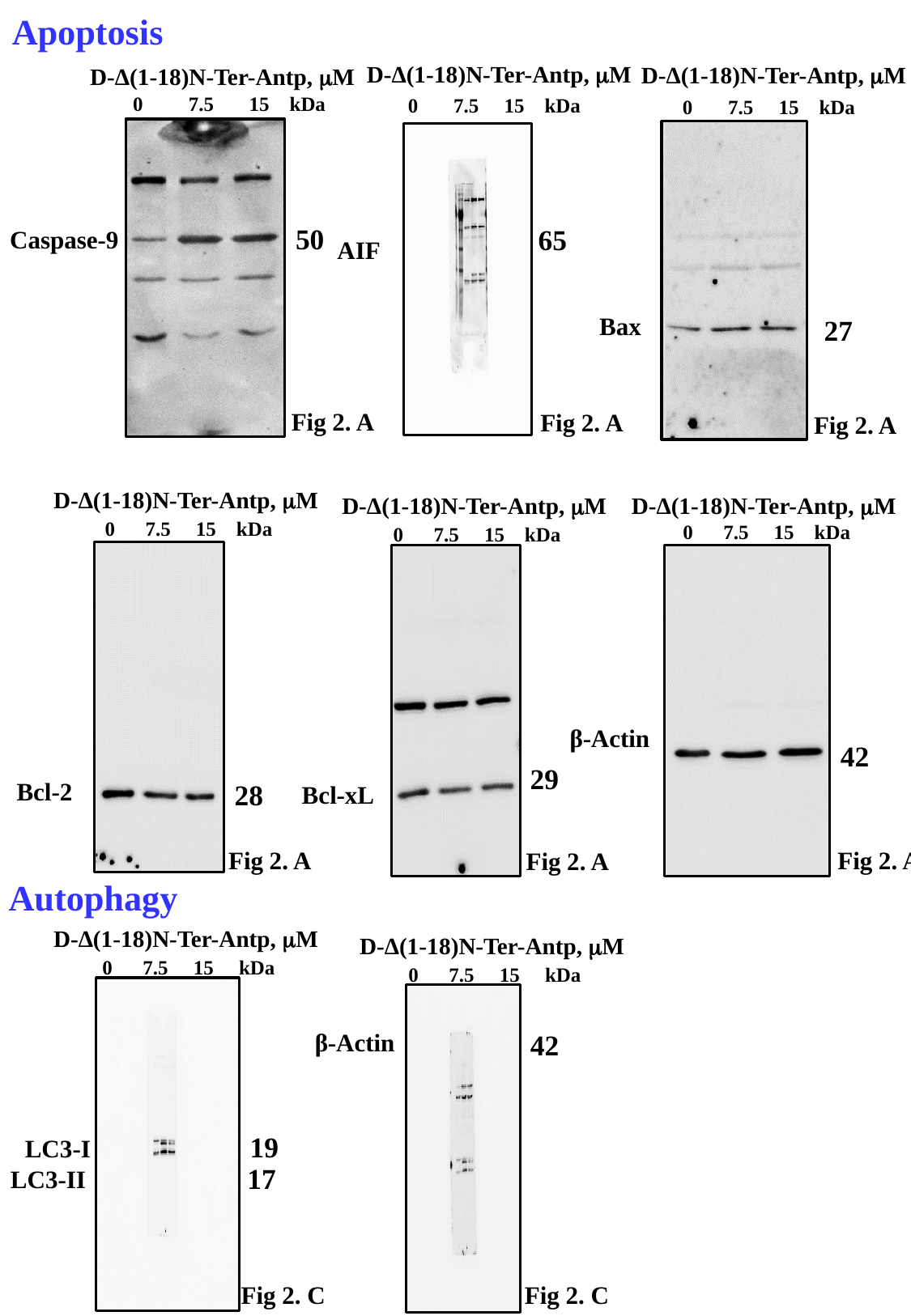

Apoptosis
D-Δ(1-18)N-Ter-Antp, mM
0 7.5 15 kDa
65
AIF
D-Δ(1-18)N-Ter-Antp, mM
0 7.5 15 kDa
Bax
27
D-Δ(1-18)N-Ter-Antp, mM
0 7.5 15 kDa
50
Caspase-9
Fig 2. A
Fig 2. A
Fig 2. A
D-Δ(1-18)N-Ter-Antp, mM
0 7.5 15 kDa
Bcl-2
28
D-Δ(1-18)N-Ter-Antp, mM
0 7.5 15 kDa
29
Bcl-xL
D-Δ(1-18)N-Ter-Antp, mM
0 7.5 15 kDa
β-Actin
42
Fig 2. A
Fig 2. A
Fig 2. A
Autophagy
D-Δ(1-18)N-Ter-Antp, mM
0 7.5 15 kDa
19
LC3-I
17
LC3-II
D-Δ(1-18)N-Ter-Antp, mM
0 7.5 15 kDa
β-Actin
42
Fig 2. C
Fig 2. C

## Slide 2
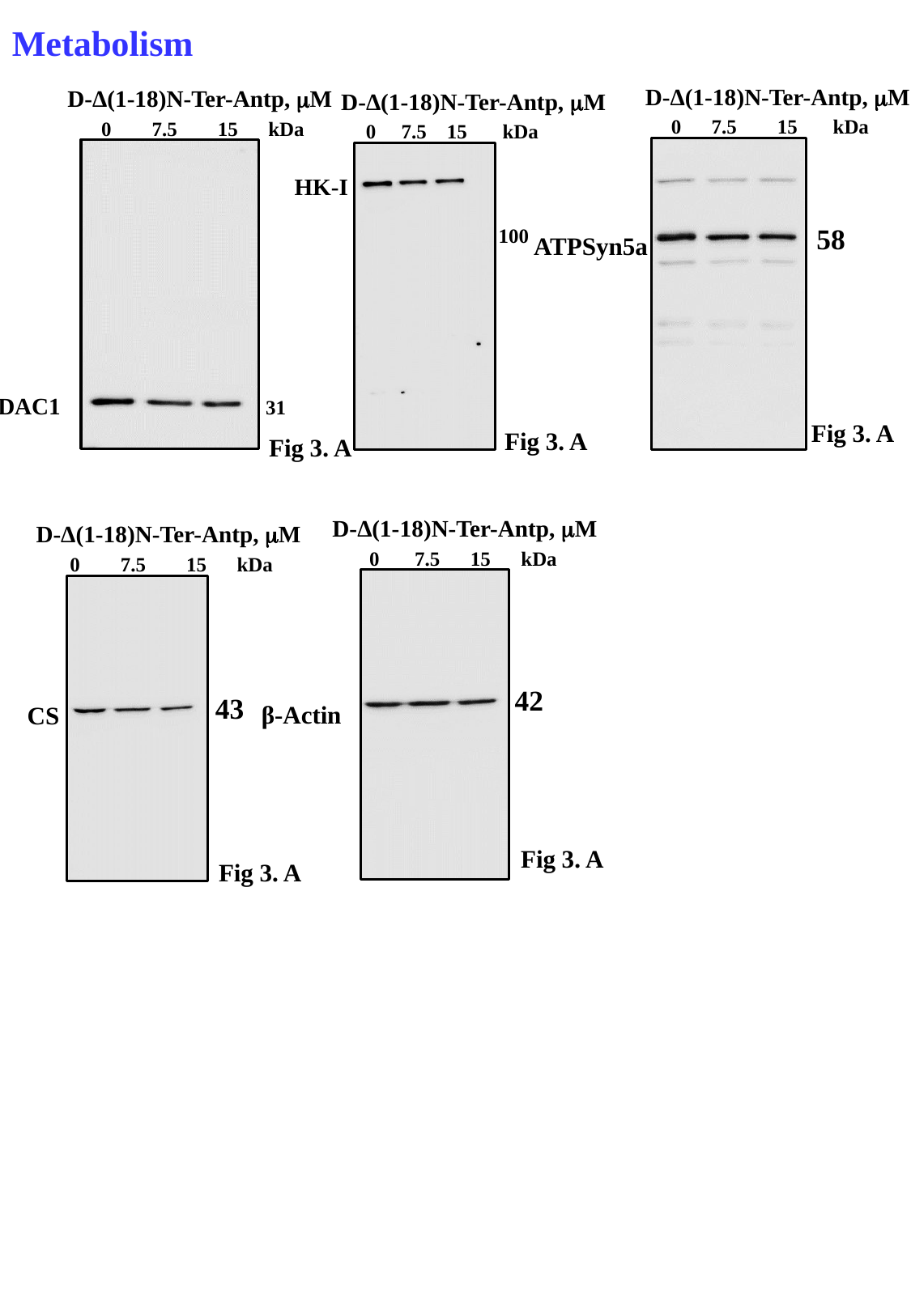

Metabolism
D-Δ(1-18)N-Ter-Antp, mM
0 7.5 15 kDa
58
ATPSyn5a
D-Δ(1-18)N-Ter-Antp, mM
0 7.5 15 kDa
VDAC1
31
D-Δ(1-18)N-Ter-Antp, mM
0 7.5 15 kDa
HK-I
100
Fig 3. A
Fig 3. A
Fig 3. A
D-Δ(1-18)N-Ter-Antp, mM
0 7.5 15 kDa
42
β-Actin
D-Δ(1-18)N-Ter-Antp, mM
0 7.5 15 kDa
43
CS
Fig 3. A
Fig 3. A

## Slide 3
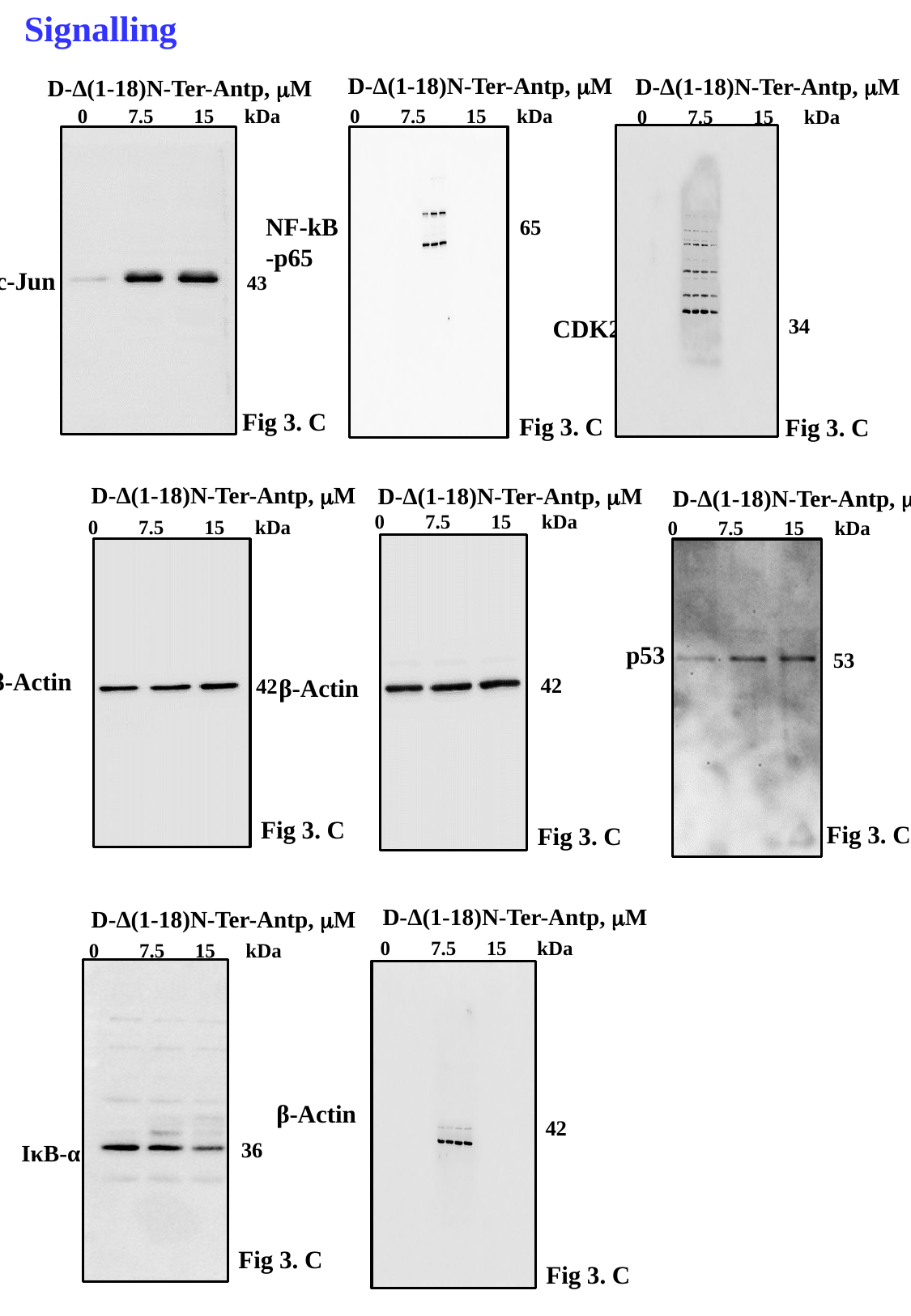

Signalling
D-Δ(1-18)N-Ter-Antp, mM
0 7.5 15 kDa
NF-kB
-p65
65
D-Δ(1-18)N-Ter-Antp, mM
0 7.5 15 kDa
34
D-Δ(1-18)N-Ter-Antp, mM
0 7.5 15 kDa
c-Jun
43
CDK2
Fig 3. C
Fig 3. C
Fig 3. C
D-Δ(1-18)N-Ter-Antp, mM
0 7.5 15 kDa
β-Actin
42
D-Δ(1-18)N-Ter-Antp, mM
0 7.5 15 kDa
β-Actin
42
D-Δ(1-18)N-Ter-Antp, mM
0 7.5 15 kDa
p53
53
Fig 3. C
Fig 3. C
Fig 3. C
D-Δ(1-18)N-Ter-Antp, mM
D-Δ(1-18)N-Ter-Antp, mM
0 7.5 15 kDa
36
IκB-α
0 7.5 15 kDa
β-Actin
42
Fig 3. C
Fig 3. C
